# Activation of the GTPase ARF6 regulates invasion of human vascular smooth muscle cells by stimulating MMP14 activity

**DOI:** 10.1038/s41598-022-13574-7

**Published:** 2022-06-09

**Authors:** Emilie Fiola-Masson, Julie Artigalas, Shirley Campbell, Audrey Claing

**Affiliations:** Department of Pharmacology and Physiology, Faculty of Medicine, Université de Montréal, Montreal, QC H3T 1J4 Canada

**Keywords:** Biochemistry, Cell biology

## Abstract

Hormones and growth factors stimulate vascular smooth muscle cells (VSMC) invasive capacities during the progression of atherosclerosis. The GTPase ARF6 is an important regulator of migration and proliferation of various cell types, but whether this small G protein can be activated by a variety of stimuli to promote invasion of VSMC remains unknown. Here, we aimed to define whether Platelet-derived growth factor (PDGF), a mitogenic stimulant of vascular tissues, and Angiotensin II (Ang II), a potent vasoactive peptide, can result in the activation of ARF6 in a human model of aortic SMC (HASMC). We demonstrate that these two stimuli can promote loading of GTP on this ARF isoform. Knockdown of ARF6 reduced the ability of both PDGF and Ang II to promote invasion suggesting that this GTPase regulates key molecular mechanisms mediating degradation of the extracellular matrix and migration. We report that PDGF-BB-mediated stimulation of ARF6 results in the activation of the MAPK/ERK1/2, PI3K/AKT and PAK pathways essential for invasion of HASMC. However, Ang II-mediated stimulation of ARF6 only promotes signaling through the MAPK/ERK1/2 and PAK pathways. These ARF6-mediated events lead to activation of MMP14, a membrane-bound collagenase upregulated in atherosclerosis. Moreover, ARF6 depletion decreases the release of MMP2 in the extracellular milieu. Altogether, our findings demonstrate that the GTPase ARF6 acts as a molecular switch to regulate specific signaling pathways that coordinate invasiveness of HASMC.

## Introduction

Vascular smooth muscle cells (VSMC) play key roles in regulating blood vessel tone and reactivity to stimuli, but also in the development of vascular diseases such as atherosclerosis. This is mainly possible because VSMC have the ability to undergo modulation of their phenotype and phenotypic behavior. In their normal differentiated state, they are quiescent and express a complement of contractile proteins to assure their basic function. Following injury to the vessel, they dedifferentiate into a synthetic phenotype that is characterized by the loss of contractile capacity, but most importantly, they gain the ability to migrate and proliferate^1^. When migrating and proliferating cells fail to switch back to the contractile phenotype after tissue reparation, due to the presence of excessive mitogens in the extracellular environment, these features are associated with the early onset of pathogenic vascular remodeling.

VSMC are mainly found in the tunica media layer, which is mostly composed of elastin and collagen fibers. As cells acquire their synthetic phenotype, basal membrane remodeling is necessary for migration to the tunica intima. By locally secreting extracellular proteases, cells can degrade the extracellular matrix, then cell motility is referred to as invasion instead of migration. This degradation is mediated by various families of proteases. One of the best characterized is the Matrix Metalloproteinases (MMP) enzymes, well known in cancer invasion. Secreted in their proactive form, they are activated by cleavage. MMP14, a membrane-type MMP, participates in vascular remodeling through its proteolytic action on collagen fibers in addition to being a main activator of secreted MMPs^2^. Known as a potent VSMC phenotypic switch factor, Platelet-Derived Growth Factor (PDGF) activates the tyrosine kinase receptor (RTK) PDGFßR to enhance MMP2 secretion, a gelatinase expressed in VSMC. Excessive activity of PDGF has been associated with several human disorders including atherosclerosis and restenosis^3,4^. The use of neutralizing antibodies against PDGF reduced VSMC invasion by about 80% in a rabbit model of atherogenesis^5^, while transgenic overexpression of this mitogen, in a porcine model, induced by eightfold intimal hyperplasia^6^. Angiotensin II (Ang II), an agonist of the G protein-coupled type 1 Ang II receptor (AT1R) and a potent vasoconstrictor, is also an inducer of MMP expression and secretion, particularly MMP2 and MMP9^7^. Ang II stimulation of VSMC is associated with the development and maintenance of neointima formation and restenosis.

Most studies describing the proteins and signaling pathways responsible for cell migration, invasion and proliferation have been performed using rodent isolated VSMC. We have shown that in VSMC from rat aortic origin, Ang II activates the small GTPase ARF6 to remodel the actin cytoskeleton and induce cell migration^8^. Ang II also increases cellular proliferation, a response we showed was regulated by ARF6^9^. ARF (ADP-ribosylation factor) proteins are monomeric GTP-binding molecules that act as molecular switches to promote downstream cellular signaling events. This family of GTPases comprises six isoforms (ARF1 to ARF6) that have specific localization and function in cells. However, ARF1 and ARF6 are the best characterized. They are key regulators of vesicle formation, membrane lipid transformation and actin remodeling^10^. Activation of ARF GTPases requires guanine-nucleotide exchange factors (GEFs) for the release of bound GDP and spontaneous loading of GTP. Inactivation requires binding of GTPase-activating proteins (GAP) to ARF-GTP to promote hydrolysis of GTP to GDP. In humans, 15 ARF GEFs have been characterized and classified into 6 families (BIG1/2, GBF, Cytohesin 1–4, BRAG1-3, EFA6A-D, FBX8). All ARF GEFs share a common Sec7 catalytic domain, but display diversity in their functions. Twenty-eight ARF GAPs are present in the human genome and divided into 10 families (ArfGAP1, ARFGAP2/3, ADAP1/2, SMAP1/2, AGFG1/2, GIT1/2, ASAP1-3, ACAP1-3, ARAP1-3, AGAP1-11). In some cases, these proteins acts as ARF-GTP effectors^11^. In rat aortic VSMC, ARF6 controls ROS formation via the activation of Rac1, NADPH oxidase, and the MAPK pathway^9^. In human cancer cells, the isoforms ARF1 and ARF6 control invasion by regulating invadopodia maturation and microvesicle formation^12–14^. ARF1 modulates the activity of the metalloprotease MMP-9 via a mechanism involving the coordination of Rho activation^12^. Although cultured VSMC of rat origin have been helpful in delineating some pathways activated by ARF6, whether these are relevant in human vascular smooth muscle cells remains to be defined.

In this study, we have examined whether ARF6 controls the invasive capacities of human VSMC and aimed at delineating the mechanism by which it may do. Our data show for the first time that PDGF-BB and Ang II promote the activation of ARF6 in human aortic SMC (HASMC) and that this GTPase is necessary for invasiveness. In this model, PDGF-BB stimulation of ARF6 regulates both activation of the MAPK and PI3K pathways, while Ang II stimulation activates only MAPK. Both stimulus however promote activation of PAK, which in turn leads to MMP14 membrane expression and activation to regulate degradation of the extracellular matrix.

## Results

### PDGF-BB and Ang II stimulation promote ARF6 activation in human aortic SMC

**Figure 1:**
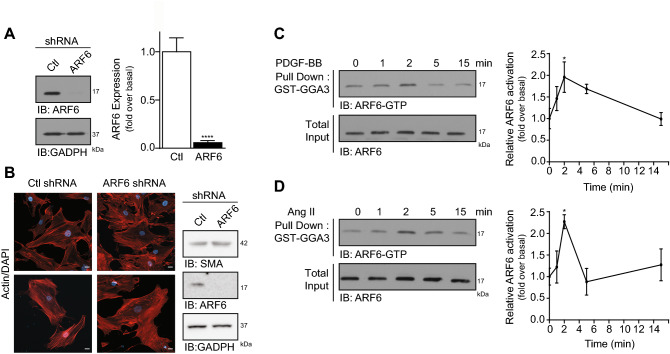
ARF6 is activated in HASMC through RTK or GPCR signaling pathway. (**A**) Cells were infected with a Ctl or ARF6 shRNA. After 72 h, cells were lysed and ARF6 expression was assessed by Western blotting using specific antibodies. Quantifications are the mean ± SEM realised on eight different experiments. **** P < 0.0001 (Student’s *t*-test). (**B**) Cells were infected as in (**A**) and after 72 h, cells were fixed and actin stained using Alexa Fluor 568 phalloidin or cells were lysed and SMA expression was assessed by Western blotting using specific antibodies (Bar = 20 µm), n = 5. (**C**, **D**) Cells were serum starved 16 h before stimulation with 25 ng/mL of PDGF-BB (**C**) or 100 nM of Ang II (**D**). They were stimulated for 0, 1, 2, 5 and 15 min. Cells were lysed and ARF6 activity was assessed in a GTPase activation assay with GST-GGA3. ARF6-GTP levels were evaluated by Western blotting using specific antibodies against ARF6. Quantifications are the mean ± SEM realised on four (**C**) or three (**D**) different experiments. *P < 0.05. (One-Way Anova).

HASMC endogenously express ARF6 and transfection of targeted shRNAs effectively knocks down protein expression by 95%, in these cells (Fig. 1A). Depletion of this GTPase does not markedly alter cellular morphology (Fig. 1B). When plated onto plastic, HASMC exhibited parallel organization of actin filament bundles observed in the contractile differentiated state although they did not fully exhibit an elongated form as they do in the in vivo setting of a normal blood vessel^15,16^. To investigate whether stimuli such as PDGF and Ang II lead to ARF6 activation in HASMC, we performed classical biochemical activation assays. As illustrated in Fig. 1C, PDGF-BB treatment resulted in a rapid and transient increase of ARF6-GTP levels, which was maximal after 2 min of stimulation and returned to basal levels 15 min post-treatment. Ang II stimulation, likewise, mediated a rapid and transient increase of active ARF6, with the maximal peak of activation at 2 min (Fig. 1D). These data demonstrate that this ARF isoform expressed endogenously can be activated in VSMC of human origin by growth factors and hormones of the cardiovascular system known to act through tyrosine kinase and G protein-coupled receptors, respectively.

### Activation of ARF6 in HASMC is necessary for invasion stimulated by PDGF and Ang II treatment

**Figure 2:**
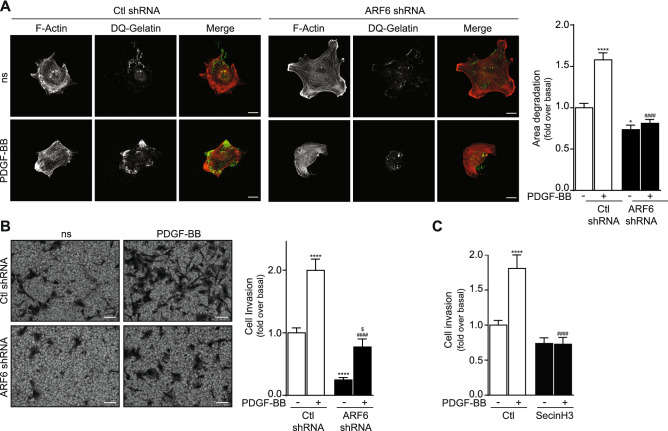
ARF6 modulates PDGF-BB-mediated invasion in HASMC. (**A**) Cells were infected with a Ctl or ARF6 shRNA and seeded onto DQ gelatin for 1 h. They were stimulated with PDGF-BB (25 ng/mL). After 6 h, cells were fixed and actin stained using Alexa Fluor 568 phalloidin. Images are representative of the degradation area of four independent experiments with 30 cells examined per condition. Quantifications are the mean ± SEM of four independent experiments. ****P < 0.0001, *P < 0.05 comparison for the basal condition, #### P < 0.0001 comparison for Ctl shRNA PDGF-BB (Two-Way Anova) (bar = 20 µm). (**B**) 100 000 cells were infected as in (**A**) and seeded into Matrigel-coated Boyden chambers for 1 h. Afterwards, cells were left untreated (non-stimulated; ns) or stimulated with PDGF-BB (25 ng/mL) for 24 h. Images are from the lower part of the membrane and are representative of five images taken per condition. Quantifications are the mean ± SEM of six independent experiments. ****P < 0.0001 are values compared to the basal condition, ####P < 0.0001 compared to Ctl shRNA PDGF-BB conditions, $ P < 0.05 compared to ARF6 shRNA ns (Two-Way Anova) (bar = 100 µm). (**C**) 100 000 cells were seeded into Matrigel-coated Boyden chambers and pretreated with DMSO (Ctl) or SecinH3 (30 µM) for 1 h. One set of cells was left untreated (non-stimulated; ns) and the other was stimulated with PDGF-BB (25 ng/mL) for 24 h. Quantifications are the mean ± SEM realised on five independent experiments. ****P < 0.0001 are values compared to basal condition, #### P < 0.0001 compared to Ctl PDGF-BB (Two-Way Anova).

To explore the importance of ARF6 in regulating the invasive capability of vascular smooth muscle cells of human origin, we used complementary experimental approaches. First, we examined the ability of agonists to promote degradation of a gelatin matrix. Plated onto a DQ-gelatin support, control and ARF6-depleted HASMC were treated with PDGF-BB for 6 h and visualization of gelatin degradation was examined by assessing the liberation of sequestered fluorochrome from the matrix. In control conditions, PDGF-BB treatment enhanced cellular invasion by 57%. Knocked down of ARF6, alone, modestly but significantly reduced the basal invasive capacity of the cells suggesting that the GTPase may be basally activated when HASMC are seeded onto an ECM. As illustrated in Fig. 2A, PDGF-BB stimulation is completely blocked when ARF6 expression is knocked down. As an alternative method, we performed Matrigel-coated transwell invasion assay. This 3D matrix is composed of type IV collagen, laminin as well as growth factors and is widely used to mimic the basement membrane. As illustrated in Fig. 2B, a 24 h stimulation with PDGF-BB-induced invasion of HASMC by twofold. Depletion of ARF6 inhibited the basal invasive capability of the cells as well as the PDGF-mediated responses.

**Figure 3:**
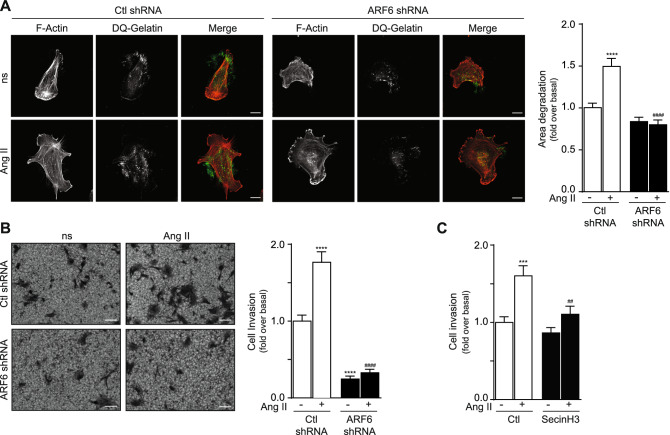
ARF6 modulates Ang II-mediated invasion in HASMC. (**A**) Cells were infected with a Ctl or ARF6 shRNA and seeded onto DQ gelatin for 1 h. They were stimulated with Ang II (100 nM). After 6 h, cells were fixed and actin stained using Alexa Fluor 568 phalloidin. Images are representative of the degradation area of five independent experiments with 30 cells examined per condition. Quantifications are the mean ± SEM of five independent experiments. ****P < 0.0001 comparison to the basal condition, ####P < 0.0001 compared to Ctl shRNA Ang II (Two-Way Anova) (bar = 20 µm). (**B**) 100,000 cells were infected as in (**A**) and seeded into Matrigel-coated Boyden chambers for 1 h. Afterwards cells were left untreated (non-stimulated; ns) or stimulated with Ang II (100 nM) for 24 h. Images are from the lower part of the membrane and are representative of five images taken per condition. Quantifications are the mean ± SEM of six independent experiments. ****P < 0.0001 are values compared to the basal condition, ####P < 0.0001 compared to Ctl shRNA Ang II (Two-Way Anova) (bar = 100 µm). (**C**) 100,000 cells were seeded into Matrigel-coated Boyden chambers and pretreated with DMSO (Ctl) or SecinH3 (30 µM) for 1 h. One set of cells was left untreated (non-stimulated; ns) and the other was stimulated with Ang II (100 nM) for 24 h. Quantifications are the mean ± SEM realised on five independent experiments. *** P < 0.001 are values compared to basal condition, ##P < 0.01 compared to Ctl Ang II (Two-Way Anova).

Treatment of HASMC with Ang II also promoted gelatin degradation. Depletion of ARF6 was an effective strategy to block agonist-mediated effects (Fig. 3A). In addition, similar results were obtained when cells were plated onto Matrigel. Ang II stimulation increased invasion in control conditions, but ARF6 depletion blocked both basal and agonist stimulated responses (Fig. 3B).

To confirm our findings, we treated HASMC with SecinH3, a biochemical inhibitor of ARF1 and 6 activation known to selective block the activity of cytohesins, a family of small guanine nucleotide exchange factors (GEFs) that regulate loading of GTP^17^. Using the transwell invasion assay, the pre-treatment of HASMC with SecinH3 (30 µM) effectively reduced PDGF-BB and Ang II-mediated invasion (Figs. 2C, 3C) further suggesting that activation of ARF proteins is necessary for HASMC invasiveness.

### PDGF-BB and Ang II promote cell invasion via an ARF6-dependent MAPK activation

**Figure 4:**
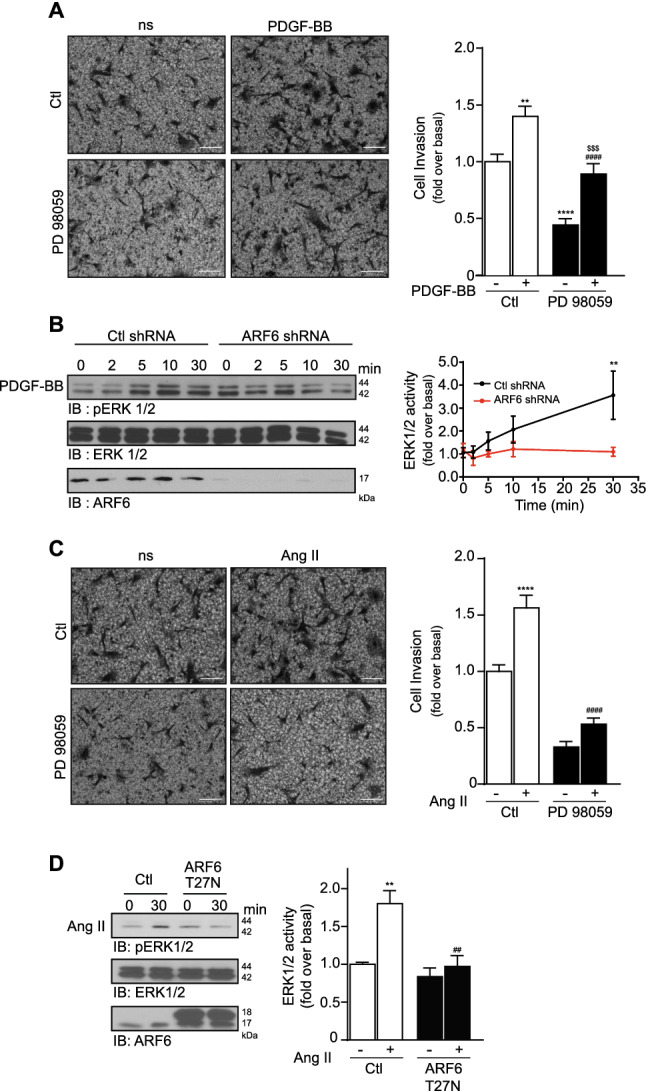
The MAPK pathway controls PDGF-BB and Ang II-dependent invasion and is regulated by ARF6. (**A**) 100 000 HASMC cells were seeded into Matrigel-coated Boyden chambers and pretreated with DMSO (Ctl) or PD98059 (25 µM) for 1 h. One set of cells was left untreated (non-stimulated; ns) and the other was stimulated with PDGF-BB (25 ng/mL) for 24 h. Images are from the lower part of the membrane and are representative of five images taken per condition. Quantifications are the mean ± SEM realised on five independent experiments. ****P < 0.0001 are values compared to basal condition, #### P < 0.0001 compared to Ctl PDGF-BB, $$$ P < 0.001 compared to PD98059 ns (Two-Way Anova) (bar = 100 µm). (**B**) Cells were infected with a Ctl or ARF6 shRNA and then serum starved 16 h before stimulation with 25 ng/mL of PDGF-BB for 0, 2, 5, 10 and 30 min. Cells were lysed and ERK1/2 activity was assessed by Western blotting using specific antibodies. Quantifications are the mean ± S.E.M. of three independent experiments. **P < 0.01 (Two-Way Anova). (**C**) Cells were seeded and pretreated as in (**A**). After 1 h, one set of cells was left untreated (non-stimulated; ns) and the other was stimulated with Ang II (100 nM) for 24 h. Images are from the lower part of the membrane and are representative of five images taken per condition. Quantifications are the mean ± SEM realised on six independent experiments. ****P < 0.0001 are values compared to basal condition, #### P < 0.0001 compared to Ctl Ang II (Two-Way Anova) (bar = 100 µm). (**D**) Cells were transfected with a Ctl or ARF6 T27N DNA plasmid using Lipofectamine 3000 according to the manufacturer’s instructions and serum starved before stimulation with Ang II for 30 min. Cells were lysed and ERK1/2 activity was assessed by Western blotting using specific antibodies. Quantifications are the mean ± SEM of three independent experiments. **P < 0.01 are values compared to basal condition, ## P < 0.01 compared to Ctl Ang II (Two-Way Anova).

To better define the molecular mechanisms by which this ARF isoform controls HASMC invasive capabilities, we aimed to identify the different downstream signaling events. In previous studies, PDGF-BB was shown to activate mitogenic cascades, which have been previously associated with VSMC invasion^18–21^. Here, we therefore first examined whether invasion was modulated by ERK1/2 pathway activation. As illustrated in Fig. 4A, cell treatment with the MEK inhibitor PD98059 (25 µM) significantly reduced basal and PDGF-BB-induced invasion establishing the importance of MAPK in this response. Next, we knocked down ARF6 expression and stimulated cells with the growth factor. In conditions where the GTPase was depleted, PDGF-BB-mediated ERK1/2 phosphorylation was reduced by 69% at 30 min (Fig. 4B). Treatment of HASMC with PD98059 also reduced basal and Ang II-dependent invasion (Fig. 4C). When we inhibited ARF6 activation by transfecting cells with the dominant negative ARF6 T^27^N mutant that mimics the GDP-bound form and sequesters ARF GEFs, MAPK activation induced by Ang II was blocked (Fig. 4D). These findings demonstrate that the MAPK pathway is a key signaling cascade activated by ARF6 during the invasion process.

### Activation of the PI3K pathway regulates PDBB-dependent invasion of HASMC

**Figure 5:**
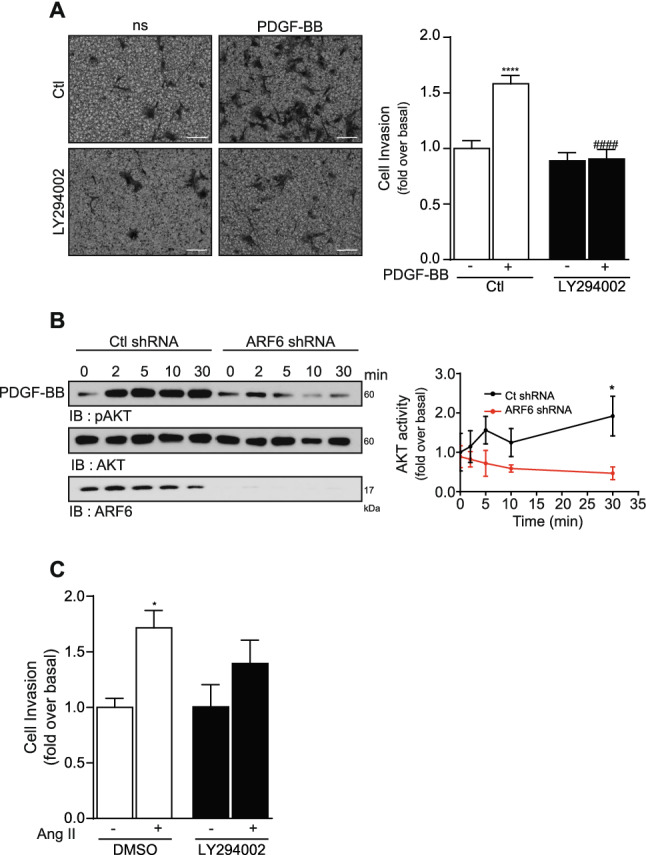
Activation of the PI3K pathway by ARF6 is important for PDGF-BB-mediated invasion. (**A**) 100 000 HASMC cells were seeded into Matrigel-coated Boyden chambers and pretreated with DMSO (Ctl) or LY294002 (25 µM) for 1 h. One set of cells was left untreated (non-stimulated; ns) and the other was stimulated with PDGF-BB (25 ng/mL) for 24 h. Images are from the lower part of the membrane and are representative of five images taken per condition. Quantifications are the mean ± SEM realised on six independent experiments. ****P < 0.0001 are values compared to basal condition, #### P < 0.0001 compared to Ctl PDGF-BB (Two-Way Anova) (bar = 100 µm). (**B**) Cells were infected with a Ctl or ARF6 shRNA and then serum starved 16 h before stimulation with 25 ng/mL of PDGF-BB for 0, 2, 5, 10 and 30 min. Cells were lysed and AKT activity was assessed by Western blotting using specific antibodies. Quantifications are the mean ± S.E.M. of three independent experiments. *P < 0.05 (Two-Way Anova). (**C**) Cells were seeded and pretreated as in (**A**). One set of cells was left untreated and the other was stimulated with Ang II (100 nM) for 24 h. Quantifications are the mean ± SEM of five independent experiments. *P < 0.05 are values compared to basal condition (Two-Way Anova).

Because both PDGF and Ang II can promote activation of the PI3K pathway in VSMC^22,23^, we aimed to verify whether this signaling axis could also be involved in invasion of HASMC. First, we studied the effect of inhibiting this pathway using the PI3K inhibitor LY294002 (25 µM). Control and treated cells were stimulated with PDGF-BB and invasion assessed using Matrigel-coated transwells. In our conditions, PI3K pathway inhibition reduced PDGF-BB-mediated invasion by 43% (Fig. 5A). However, treatment with this inhibitor did not affect Ang II-mediated invasion (Fig. 5C). We next examined whether depletion of ARF6 affected PDGF-BB stimulated PI3K activation. We found that when cells were infected with the ARF6 shRNA, AKT phosphorylation was reduced by 76% (Fig. 5B).

Altogether, these findings suggest that PDGF-BB can activate ARF6, which in turn activates the PI3K signaling cascade to promote invasion of human VSMC.

### The PAK pathway controls PDGF-BB and Ang II-mediated invasion

**Figure 6:**
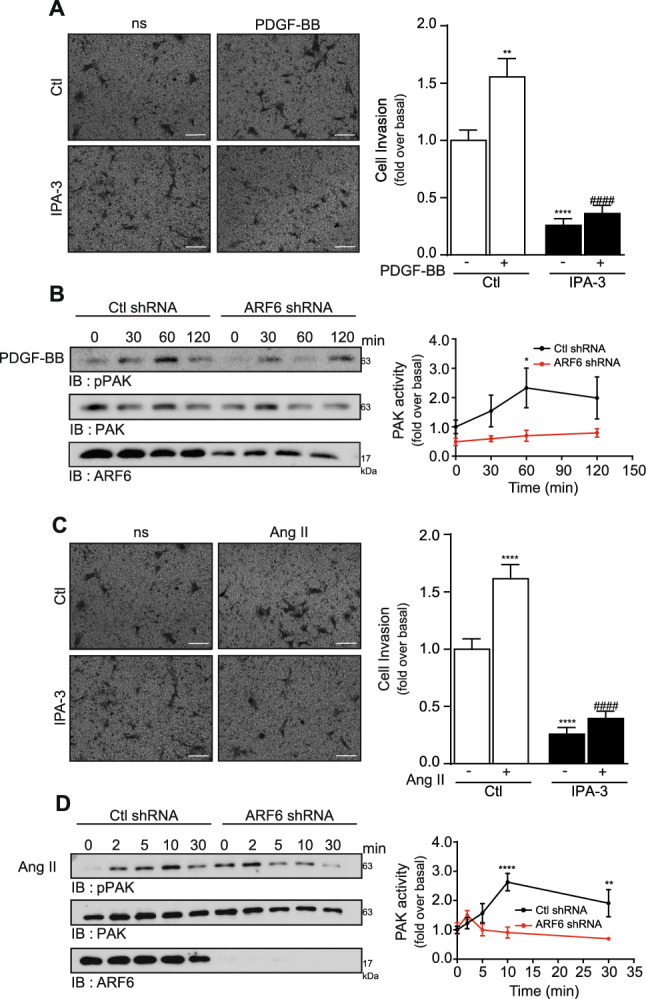
Activation of PAK is important for PDGF-BB and Ang II-induced invasion. (**A**) 100 000 HASMC cells were seeded into Matrigel-coated Boyden chambers and pretreated with DMSO (Ctl) or IPA-3 (10 µM) for 1 h. One set of cells was left untreated (non-stimulated; ns) and the other was stimulated with PDGF-BB (25 ng/mL) for 24 h. Images are from the lower part of the membrane and are representative of five images taken per condition. Quantifications are the mean ± SEM realised on four independent experiments. ****P < 0.0001, **P < 0.01 are values compared to basal condition, #### P < 0.0001 compared to Ctl PDGF-BB (Two-Way Anova). (**B**) Cells were infected with a Ctl or ARF6 shRNA and then serum starved 16 h before stimulation with 25 ng/mL of PDGF-BB for 0, 30, 60 and 120 min. Cells were lysed and PAK activity was assessed by Western blotting using specific antibodies. Quantifications are the mean ± S.E.M. of five independent experiments. *P < 0.05 (Two-Way Anova). (**C**) 100 000 HASMC cells were seeded and pretreated as in (**A**). One set of cells was left untreated (non-stimulated; ns) and the other was stimulated with Ang II (100 nM) for 24 h. Images are from the lower part of the membrane and are representative of five images taken per condition. Quantifications are the mean ± SEM realised on four independent experiments. ****P < 0.0001 are values compared to basal condition, #### P < 0.0001 compared to Ctl Ang II (Two-Way Anova). (**D**) Cells were infected with a Ctl or ARF6 shRNA and then serum starved 16 h before stimulation with Ang II 100 nM for 0, 2, 5, 10 and 30 min. Cells were lysed and PAK activity was assessed by Western blotting using specific antibodies. Quantifications are the mean ± S.E.M. of five independent experiments. ****P < 0.0001, **P < 0.01 (Two-Way Anova).

To further gain insights into the mechanisms by which ARF6 regulates HASMC invasiveness, we investigated the role of other signaling events. It was reported that ARF6 can interact with diverse effectors to promote remodeling of the cytoskeleton, a step necessary for invasion^24,25^. Considering that PAK proteins are involved in cytoskeletal protein rearrangement and rat VSMC migration^26^, we examined whether this pathway could modulate PDGF-BB or Ang II-mediated cellular invasion. As illustrated in Fig. 6A, pretreatment of HASMC with the PAK Group 1 inhibitor, IPA-3 (10 µM), significantly reduced basal cellular invasive capabilities as well as PDGF-BB and Ang II-stimulated invasion suggesting an important role for PAK activation in this response. Next, we infected cells with either control or ARF6 shRNA and assessed PAK phosphorylation. PDGF-BB stimulation promoted activation of this kinase with a peak at 60 min. PAK phosphorylation was decreased in conditions where we depleted cells of ARF6 (Fig. 6B). Likewise, treatment of cells with IPA3 affected basal and Ang II-mediated invasion (Fig. 6C). To verify whether ARF6 could act as a molecular switch to modulate PAK activity, we stimulated control and ARF6-depleted cells with Ang II and assessed PAK phosphorylation. Inhibition of ARF6 expression reduced Ang II-mediated PAK activation (Fig. 6D).

These results suggest that PAK is a key signaling kinase that acts to mediate Ang II and PDGF-induced invasion in our cellular model and that ARF6 is a key regulator of this signaling pathway.

### Extracellular proteases such as MMP2 and MMP14 mediates ARF6-dependent ECM degradation and invasion

**Figure 7:**
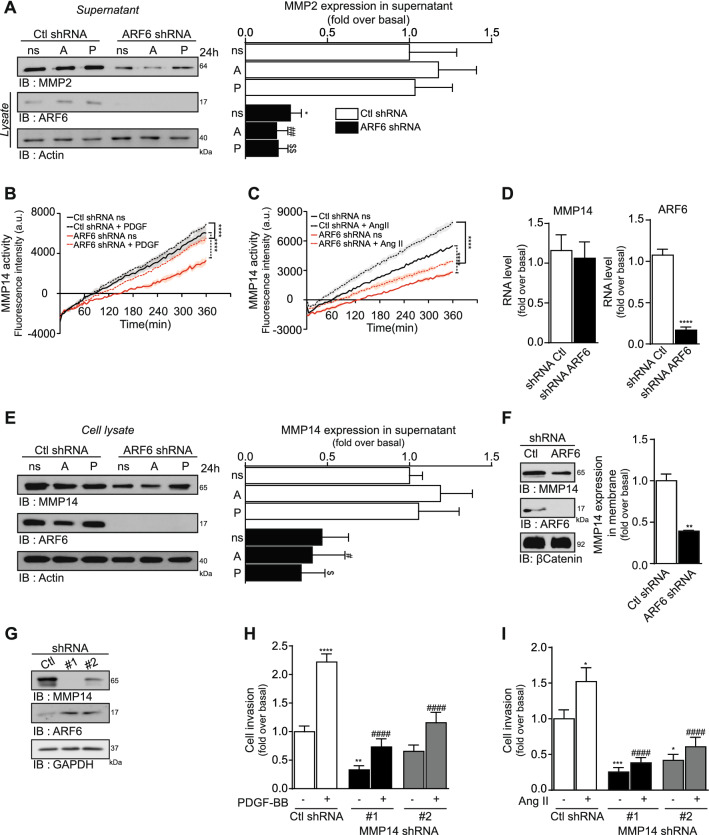
MMP14, essential for HASMC invasion, is regulated by ARF6. (**A**) Cells were infected with a Ctl or ARF6 shRNA, serum starved, and stimulated with Ang II (100 nM) (A) or PDGF-BB (25 ng/mL) (P) for 24 h. Supernatants were collected and concentrated. MMP2 expression was assessed by Western blotting using specific antibodies. Quantifications are the mean ± S.E.M. of six independent experiments. *P < 0.05 value compared to Ctl ns, ##P < 0.01 compared to Ctl Ang II, $$ P < 0.01 compared to Ctl PDGF-BB (Two-Way Anova). (**B**, **C**) Cells were infected as in (**A**). Before PDGF-BB stimulation (**B**) or Ang II (**C**) for 6 h, medium was changed for L-15 with MMP14 fluorogenic substrate (10 µM). The fluorescence intensity was measured at 328/400 nm every 5 min. Quantifications are the mean ± S.E.M. of duplicates of one experiment, representative of 3 independent experiments, ****P < 0.0001 (One-Way Anova). (**D**) Cells were infected as in (**A**) and total RNA was extracted with Qiagen RNeasy Kit according to the manufacturer’s instructions. Quantifications are the mean ± S.E.M. of three independent experiments. ****P < 0.0001 (Student’s *t*-test) (**E**) Cells were infected and stimulated as in (**A**). Cells were lysed and MMP14 expression was assessed by Western blotting using specific antibodies. Quantifications are the mean ± S.E.M. of three independent experiments. #P < 0.05 value compared to Ctl Ang II, $P < 0.05 compared to Ctl PDGF-BB (Two-Way Anova) (**F**) Cells were infected as in (**A**) and membrane proteins were isolated by ultracentrifugation. Quantifications are the mean ± S.E.M. of three independent experiments. **P < 0.01 (Student’s *t*-test). (**G**) Cells were infected with Ctl, MMP14-1 (#1) or MMP14-2 (#2) shRNA and lysed. Blots are representative of six independent experiments. (**H, I**) Cells were infected with a Ctl or two MMP14 shRNA (#1 or #2) and seeded into Matrigel-coated Boyden chambers for 1 h. Afterwards cells were left non-stimulated (ns) or stimulated with PDGF-BB (**H**) or Ang II (**I**) for 24 h. Quantifications are the mean ± SEM of six independent experiments. (**H**)****P < 0.0001, **P < 0.01 comparison to the basal condition, ####P < 0.0001 compared to Ctl PDGF-BB (Two-Way Anova). (**I**) ***P < 0.001, *P < 0.05 comparison to the basal condition, ####P < 0.0001 compared to Ctl Ang II (Two-Way Anova).

For invasion to take place, ECM must be degraded by cell secreted proteases. In atherosclerosis, MMP2 and MMP9 are known to be overexpressed and act as key regulators of ECM remodeling^27,28^. These MMPs also promote invasion in rodent VSMC^29,30^. To determine their role in our model, we aimed to verify whether their function could be altered in the absence of ARF6. As illustrated in Fig. 7A, knockdown of ARF6 reduced by 73% levels of MMP2 in the supernatant. However, MMP9 was not detected in our cellular model (mRNA, data not shown) suggesting that this enzyme is not responsible for HASMC invasion. Because MMP2 activation is dependent upon cleavage of its proform by MMP14^2^, a membrane-associated MMP, we aimed to determine if the downregulation of MMP2 levels in the supernatant was correlated with modification of MMP14 activity. As illustrated in Fig. 7B, MMP14 activity was increased following PDGF-BB stimulation. ARF6 knockdown significantly decreased both basal and growth factor-stimulated MMP14 activity. Similarly, Ang II treatment of the cells enhanced MMP14 activity and knockdown of ARF6 blocked this response (Fig. 7C). To better define the mechanisms by which ARF6 might regulate activity of this enzyme, we investigated whether the GTPase acted to control transcription of the MMP14 gene. As shown in Fig. 7D, MMP14 mRNA levels were similar in control and ARF6 shRNA infected cells. However, total MMP14 protein level was reduced by more than 50% in ARF6-depleted cells (Fig. 7E). Specifically, depletion of ARF6 significantly reduced the presence of MMP14 at the plasma membrane (Fig. 7F). These results suggest that ARF6 controls MMP14 enzymatic activity by regulating the very presence of the latter at the cell surface, therefore affecting the levels of MMP2. To confirm that MMP14 was indeed a key MMP regulating HASMC invasion, we knocked down its expression using two different shRNA sequences. These tools reduced MMP14 protein expression by 97% and 63%, respectively, without any specific effect on ARF6 expression (Fig. 7G). As illustrated in Fig. 7H,I, basal and agonist stimulated invasion was reduced when cells were infected with either MMP14 shRNA sequences (Fig. 7I).

Altogether, these results suggest that MMP14 is a key protease modulating invasion of HASMC and that ARF6 acts upstream to control its activity.

## Discussion

The acquisition of invasive properties is essential for VSMC during the development of vascular diseases such as atherosclerosis. Cells that exhibit a synthetic phenotype need to degrade the extracellular matrix by proteolysis in order to migrate and form the intimal layer of the diseased vessel. Numerous signaling events are involved in this process, but our complete understanding of the signaling cascades leading to cell invasiveness, in human VSMC, has yet to be fully defined. Here, we report that the small GTPase ARF6 is an upstream molecular switch activated by receptor agonists and stimuli of the cardiovascular system, Ang II and PDGF-BB. These factors are known inducers of VSMC phenotypic switching^31,32^. By coordinating the classical signaling pathways such as MAPK, PI3K/AKT, and PAK, ARF6 regulates activation of MMP14 and MMP2 responsible for ECM degradation, allowing cells to migrate across tissue.

In recent years, we demonstrated that ARF proteins were a key regulator of phenotypic switching and key physiological responses such as migration and proliferation using rat aortic VSMC^8,9^. Although this model is widely used, whether our observations could be translated into the human pathophysiology remained a concern. Here, using primary aortic smooth muscle cells of human origin, we show for the first time that ARF6 controls stimuli-mediated cellular invasion. By establishing that both GPCR and RTK-dependent activation of ARF6 is required for HASMC, we propose that signaling through this GTPase may be a broadly used mechanism. The function of ARF6 was assessed using complementary approaches such as knockdown by shRNA, biochemical ARF inhibition and overexpressed dominant negative mutants. Furthermore, cellular invasion was investigated using a gelatin matrix (confocal microscopy) and Matrigel (Transwell assay) to allow us to assess cellular movement as well as matrix degradation. Our data clearly show that ARF6 inhibition by reduced expression or blockade of its activation reduced HASMC invasion.

The role of ARF proteins, in regulating HASMC invasiveness, is reminiscent of the role this GTPase plays in tumor cells^33^. Both ARF1 and ARF6 are located in invadopodia as well as in shedding microvesicles, the key structures regulating cellular invasion through the release of proteolytic activity^12,13^. VSMC can also invade their surrounding through the formation of actin structures, called podosomes, that release proteases degrading ECM^34^. However, these structures are mostly reported in VSMC of mouse or rat origin^35,36^. In triple negative breast cancer cells (TNBC), we showed that ARF1 controlled mainly the activation of MMP9 and MMP2 through FAK^37^. ARF6, together with it effectors JIP3/4, regulated MMP14 exocytosis^38^. Although the activity of metalloproteases is highly conserved, the mechanisms by which ARF proteins regulate key signaling events leading to their activation may be different. In HASMC, PAK is the key enzyme regulating MMP14 and MMP2, in contrast to FAK in TNBC. MMP14, a membrane MMP, is an important pericellular proteinase (collagenase) and regulator of MMP2 associated with vascular remodeling during pathologies such as neointimal formation^39^. In HASMC, reduced MMP14 enzymatic activity in cells depleted of ARF6 is likely to be associated with reduced expression at the plasma membrane as suggested by our findings.

To better define the signaling events regulated by ARF6 during invasion, we investigated the contribution of the MAPK/ERK1/2 and PI3K/AKT pathways. First, we demonstrated that PDGF-BB and Ang II stimulation did not require identical ARF6-dependent signaling events although inhibitors of both these pathways blocked invasion in our cellular model. Both factors require ARF6-mediated activation of MAPK while only PDGF-BB additionally involved PI3K/AKT. These findings suggest that ARF6 may regulate a subset of events depending on the nature of the stimuli. Further, our use of alternative tools that altered expression and/or activation of ARF6 resulted in decrease or not of the basal invasive capacities of HASMC. When we depleted cells of ARF6, both basal and agonist-mediated effects were inhibited. The ability of HASMC to exhibit invasive activity in absence of our stimuli is likely due to the components of the ECM such as macromolecules (gelatin, laminin, collagen) and growth factors that do engage membrane receptor activation and intracellular signaling cascades. Matrigel, namely, is a complex extracellular matrix (ECM) mixture secreted by Engelbreth–Holm–Swarm mouse sarcoma cells with an approximate composition of 60% laminin, 30% collagen IV and 8% entactin as well as other undefined ECM components and growth factors^40^. As an alternative approach to target ARF6, we used a GTP-bound mimic mutant of the GTPase or a biochemical inhibitor. These tools blocked GTP-loading on the GTPase and were only effective in reducing stimuli-dependent responses. ARF6T^27^N can form a complex with ARF GEFs, at the membrane, to prevent endogenous GTPase activation accounting for its dominant-negative effect. However, ARF6 remains present. Treatment of the cells with SecinH3 blocks only the ability of the cytohesin family of ARF GEFs to promote loading of GTP on ARF6. Other exchange factors may be responsible for a portion of activation of this ARF isoform, namely in the context of basal invasion. Although no ARF GEF has been identified as essential for VSMC invasion, several studies have demonstrated roles for GEP100 in cancer cell invasion^41–44^. Cytohesin and other GEFs likely contribute to ARF6 activation.

Our experiments also highlighted the key role of PAK in agonist-mediated invasion of HASMC. This kinase is an effector for cytoskeleton reorganisation, a key step of the invasion process. Interestingly, PAK pathways have been linked to invasion of cancer cells^45^. Considering that PAK is activated by Rac1 and Cdc42, two members of the Rho family of GTPases and that the latter are modulated by ARF6 in rat VSMC, this enzyme was a good candidate for invasion regulation in our model. We showed that ARF6 modulated PAK phosphorylation, an event necessary for HASMC invasion.

Altogether, our study has contributed to elucidating the role ARF6 plays in invasion of human primary VSMC (Supplementary Fig. 1). This ARF isoform can be activated following RTK or GPCR stimulation and regulate the activation of different signaling pathways that will promote invasion. Our findings shed light on the molecular mechanisms implicated in this cellular response induced by ARF6 activation. A better understanding of the key regulators of HASMC invasion will help us identify potential new therapeutic targets for the treatment of diseases of, namely, the vascular wall characterized by aberrant SMC function.

## Methods

### Reagents and antibodies

BD Matrigel Matrix was purchased from BD Science (Bedford, MA). Alexa Fluor 568-phalloidin, DQ-Gelatin and puromycin were purchased from Invitrogen (Burlington, ON). Protein G PLUS-agarose beads, antibodies against ARF6 (3A-1), anti-ERK1/2 and anti-GAPDH were from Santa Cruz Biotechnology (Santa Cruz, CA). Anti-phospho-ERK1/2, anti-AKT, anti-phospho-AKT, anti-phosphoPAK, anti-PAK, anti-MMP2 and anti-Actin were purchased from Cell Signaling (Danvers, MA). PDGF-BB was obtained from Fitzgerald Industries Int’l (Acton, MA). SecinH3 was purchased from Abcam Biochemicals (Cambridge, MA). LY294002 was purchased from Cayman Chemical (Ann Arbor, MI). All other products were obtained from Sigma Aldrich (Oakville, ON).

### shRNA

Plasmids were purchased from the MISSION shRNA Library, Sigma Aldrich (ARF6: TRCN0000380270, MMP14-1: TRCN0000050855, MMP14-2: TRCN0000050857, Ctl: SHC016). Lentiviruses containing the shRNA were generated using HEK293T cells transfected with the shRNA plasmid and the psPax.2 and pMD2.G packaging plasmids using a calcium phosphate mix (HBS 2X: 50 mM HEPES, pH 7.1, 280 mM NaCl, 1.5 mM Na_2_HPO_4_, mixed with 2.5 mM CaCl_2_).

### Cell culture, shRNA lentivirus infection and transfection

Human Aortic Smooth Muscle Cells (HASMC) were obtained from ScienCell Research Laboratories (Carlsbad, CA), cultured in smooth muscle cell medium (ScienCell) according to the manufacturer’s instructions and maintained in a humidified incubator with 5% CO_2_ at 37 °C. All experiments were performed between passages 4–8. HASMC were infected in the presence of polybrene (8 μg/ml) with untargeted (control), ARF6 shRNA or MMP14 shRNA lentiviruses. Stable clones were selected using puromycin (5 μg/ml) and cells were used for experiments 72 h post-infection. HASMC were transfected with ARF6T^27^N pcDNA 3.0 or empty plasmid (Ctl) in the presence of Lipofectamine 3000 according to the manufacturer’s instructions. Cells were used for experiments 72 post-transfection.

### Cell invasion assay

For all experiments with control, ARF6 shRNA or MMP14 shRNA, 100 000 HASMC were infected for 72 h and then serum starved for 16 h before being seeded into Boyden chambers (24-well inserts with 8-μm pore coated with Matrigel). One hour after plating, cells were stimulated with PDGF-BB (25 ng/ml) or Ang II (100 nM) in the lower chamber for 24 h. Cells were fixed and stained using crystal violet (0.1% in 20% MeOH: overnight), the membranes were washed three times with H_2_O, and cells were removed from the upper chamber with cotton tipped applicators (Innovatek, Vancouver, BC). Pictures of five different fields of the inferior side of the membranes were taken, and the average number of invading cells was quantified manually for each condition. For conditions with inhibitors, cells were pre-treated with vehicle (DMSO 0.1%), PD98059 (25 μM), LY29402 (25 µM) or IPA-3 (10 µM) for 1 h before performing the experiments as described above.

### GST pulldown assay—ARF6 activation assay

HASMC were serum starved for 16 h and stimulated for the indicated times with PDGF-BB and Ang II. Cells were harvested in lysis buffer E (pH 7.4, 50 mM Tris–HCl, 1% Nonidet P-40, 137 mM NaCl, 10% glycerol, 5 mM MgCl_2_, 20 mM NaF, 1 mM NaPPi, 1 mM Na_3_VO_4_, and protease inhibitors). Samples were spun for 10 min at 10,000 × g at 4 °C. GST-GGA3 fusion proteins coupled to glutathione-Sepharose 4B beads were added to each tube, and samples were rotated at 4 °C for 1 h^46^. Beads were washed, and proteins were eluted into 20 μl of SDS-sample buffer containing 5% β-mercaptoethanol by heating to 65 °C for 15 min and resolved on 14% SDS-PAGE. Western blot analysis was done using a specific anti-ARF6 antibody.

### Western blotting

HASMC were harvested, and total soluble proteins were run on SDS-PAGE gels and transferred onto nitrocellulose membranes. For MMP2 experiments, the supernatant was collected and concentrated with Amicon 15-30 K columns, and then total proteins were run on a gel and transferred onto membranes. Isolation of membrane proteins was previously described^47^. The membranes were cut and blotted for relevant proteins using specific primary antibodies (as described for each experiment). Secondary antibodies were HRP-conjugated and the chemiluminescence reaction was triggered using the Amersham ECL Prime Western detection reagent. Membranes were exposed to autoradiography films, which were scanned using a Canon scanner. Alternatively, membranes were digitalized using GE LAS 4000 mini. Quantification of the digital images obtained was performed using ImageJ (version 1.52a). For Figs. 1B, 6B and 7A,G, images were processed according to digital image and integrity policies of Nature. Increase in contrast was applied equally across the entire image for a better visualization.

### Matrix degradation assay

Sterile glass coverslips were coated with a DQ-gelatin solution (50 µg/ml DQ-gelatin, 2% bovine gelatin, 2% D-sucrose in PBS) for 90 min, at room temperature and in the dark. Coverslips were rinsed in PBS and left in media without serum. Cells were then plated onto coverslips and incubated for 1 h before stimulation with PDGF-BB or Ang II. Six hours post-stimulation, cells were fixed with a 4% paraformaldehyde solution, for 10 min, at room temperature, and then permeabilized with 0.5% Triton X-100 solution for 10 min. After BSA blocking, they were labeled with Alexa-Fluor 568 phalloidin. Coverslips were mounted onto slides with Aqua-Mount and analyzed using a Zeiss confocal microscope LSM800.

### MMP14 activity assay

HASMC were infected with control, ARF6 or MMP14-1 directed shRNAs as described above. 25 000 cells were plated into a black 96-well plate and then serum starved overnight. Fluorogenic MMP14-substrate (Sigma-Millipore) was added to L15 media (Wisent, St-Bruno Qc) at a final concentration of 10 µM in presence of PDGF-BB (25 ng/ml), Ang II (100 nM) or left unstimulated (ns). Fluorescence was measured at 328/400 nm every 5 min for 6 h, using the Synergy H1 Plate Reader (BioTek). Experiments were performed in duplicate. Fluorescence intensity was determined by subtracting the background from each condition.

### Statistical analysis

Statistical analyses were conducted with GraphPad Prism software version 7.0 (GraphPad PRISM, San Diego, CA) using the Student’s *t*-test, a one-way analysis of variance with post hoc Tukey corrections or a two-way analysis. A P < 0.05 was considered statistically significant.

## Supplementary Information

Supplementary Legends.

Supplementary Figure S2.

Supplementary Information 3.

## Data Availability

The datasets generated during and/or analysed during the current study are available from the corresponding author on request.
