# Supplementary material for: Activation of the GTPase ARF6 regulates invasion of human vascular smooth muscle cells by stimulating MMP14 activity
Source: Sci Rep. 2022 Jun 9;12:9532. doi: 10.1038/s41598-022-13574-7 (PMC9184495; doi:10.1038/s41598-022-13574-7)
Supplement: Supplementary file 1 — Supplementary Legends. [file 41598_2022_13574_MOESM1_ESM.docx]

LEGEND Supplemental figure

Figure 1. Schematic representation of stimulation-mediated HASMC invasion. Circulating PDGF-BB and Ang II activate specific receptors expressed on the plasma membrane of HASMC. Through proximal activation of factors such as G proteins, ßarrestin, adaptor proteins and ARF GEF, these receptors can promote the activation of the small GTP-binding protein ARF6. Activated ARF6, in turn, acts as a molecular switch to activate in time and in place, different signaling pathways such as the MAPK/ERK1/2, PI3K/AKT or PAK, depending on the nature of the stimuli. This leads to relocalisation of MMP14 at the membrane, which mediates ECM degradation directly or through regulation of MMP2 activation.
