# Supplementary material for: Activation of the GTPase ARF6 regulates invasion of human vascular smooth muscle cells by stimulating MMP14 activity
Source: Sci Rep. 2022 Jun 9;12:9532. doi: 10.1038/s41598-022-13574-7 (PMC9184495; doi:10.1038/s41598-022-13574-7)
Supplement: Supplementary file 3 — Supplementary Information 3. [file 41598_2022_13574_MOESM3_ESM.docx]

Full Gel

**Fig. 1A**


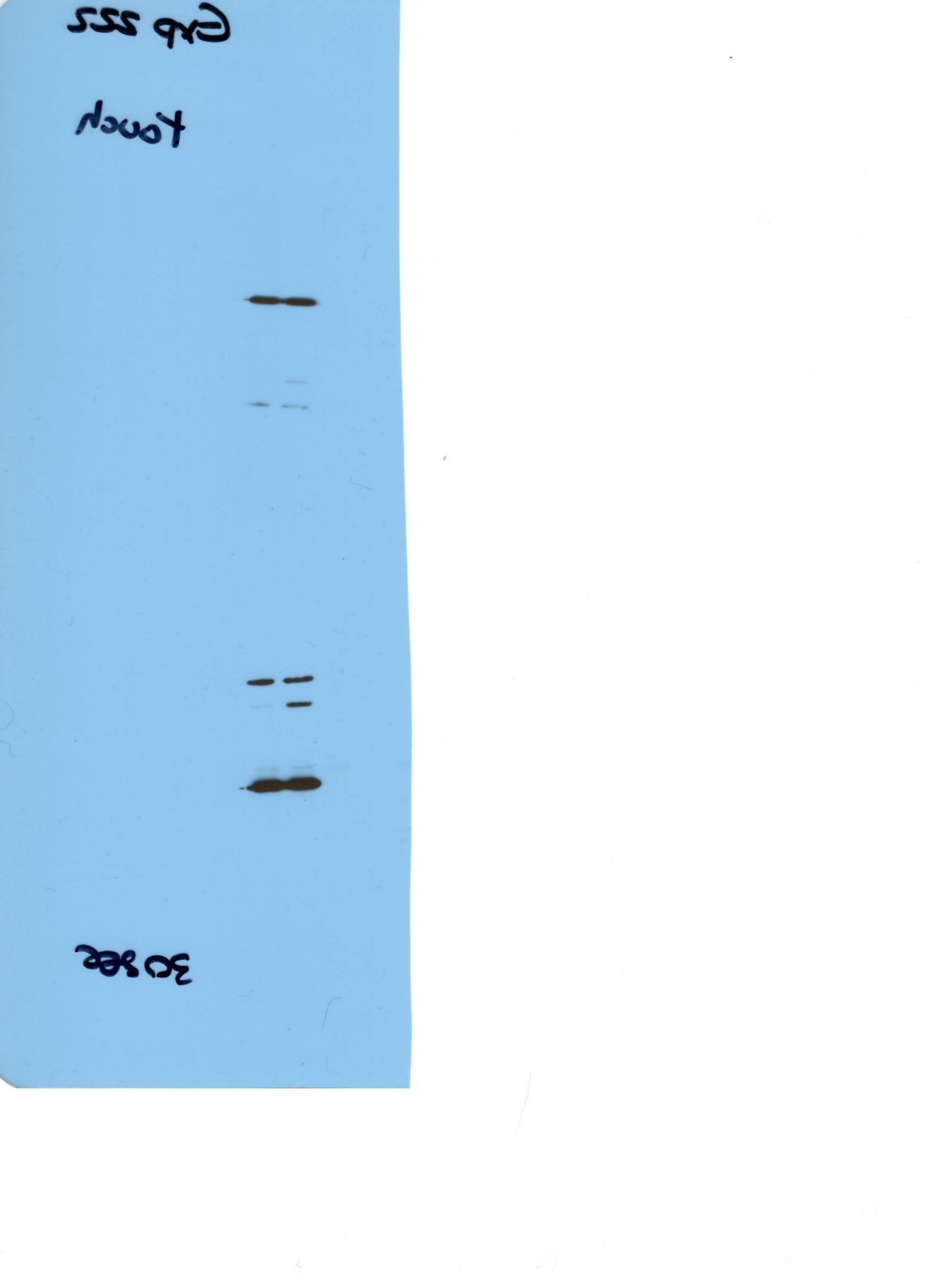

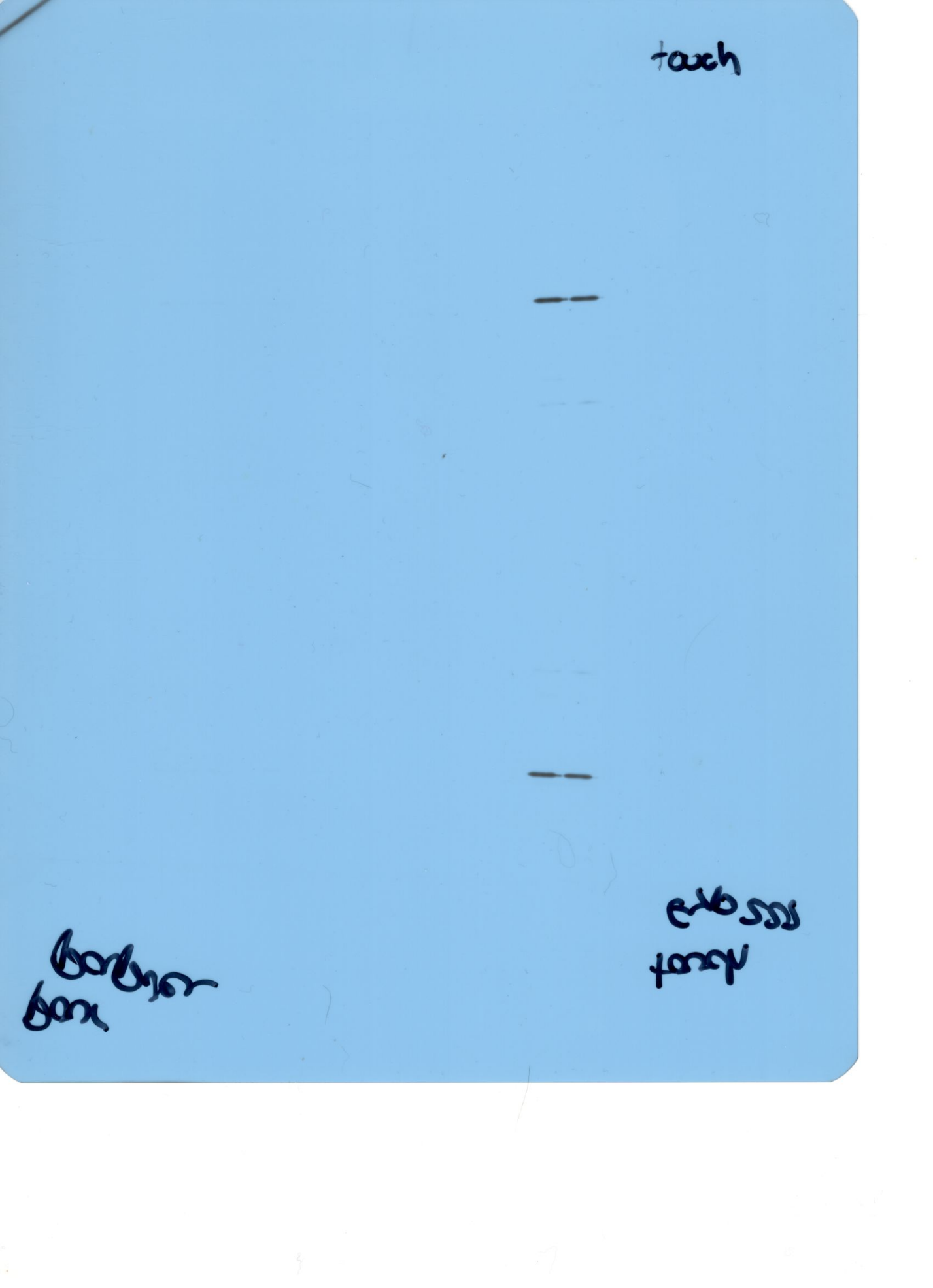


47

47

Anti-ARF6

17

17

33

33

Anti-GAPDH

**Fig. 1B**

Anti-SMA

50

**
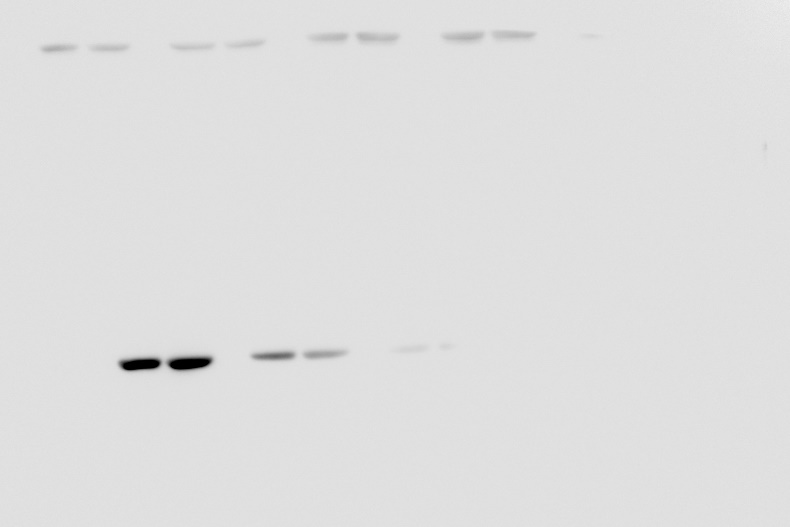
**

Gel 2

Gel 1

50

37

37

Anti-ARF6


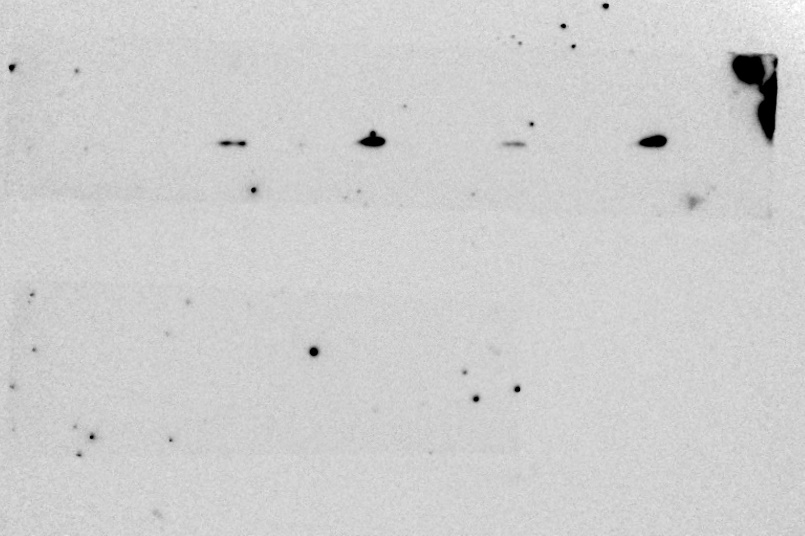


25

20

Anti-GAPDH


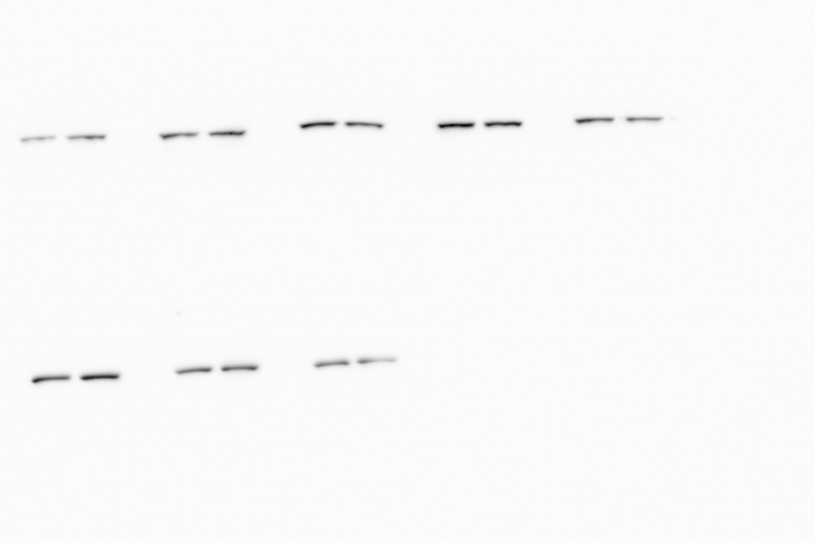


Gel 1

Gel 2

37

37

50

50

**Fig. 1C**


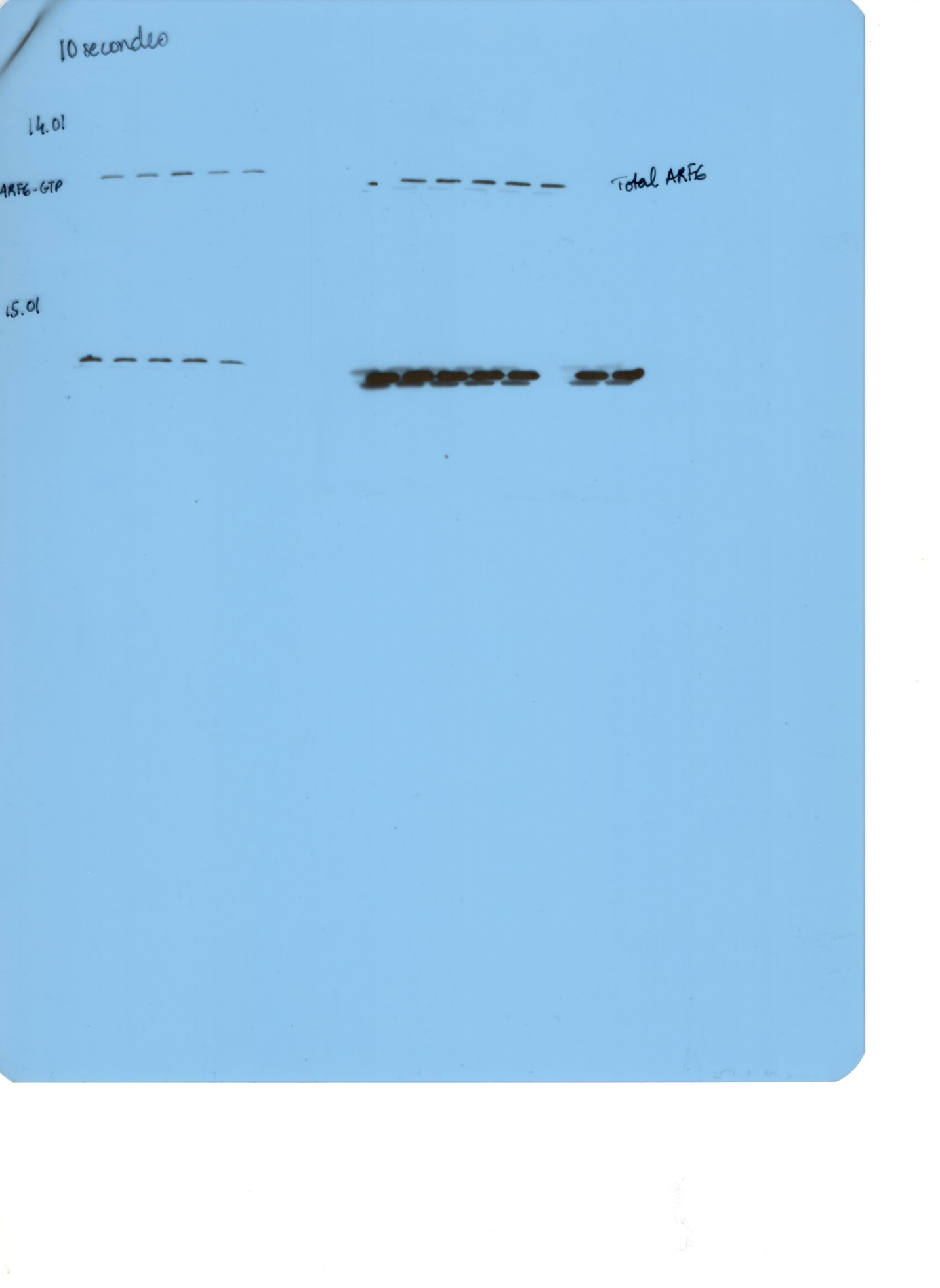

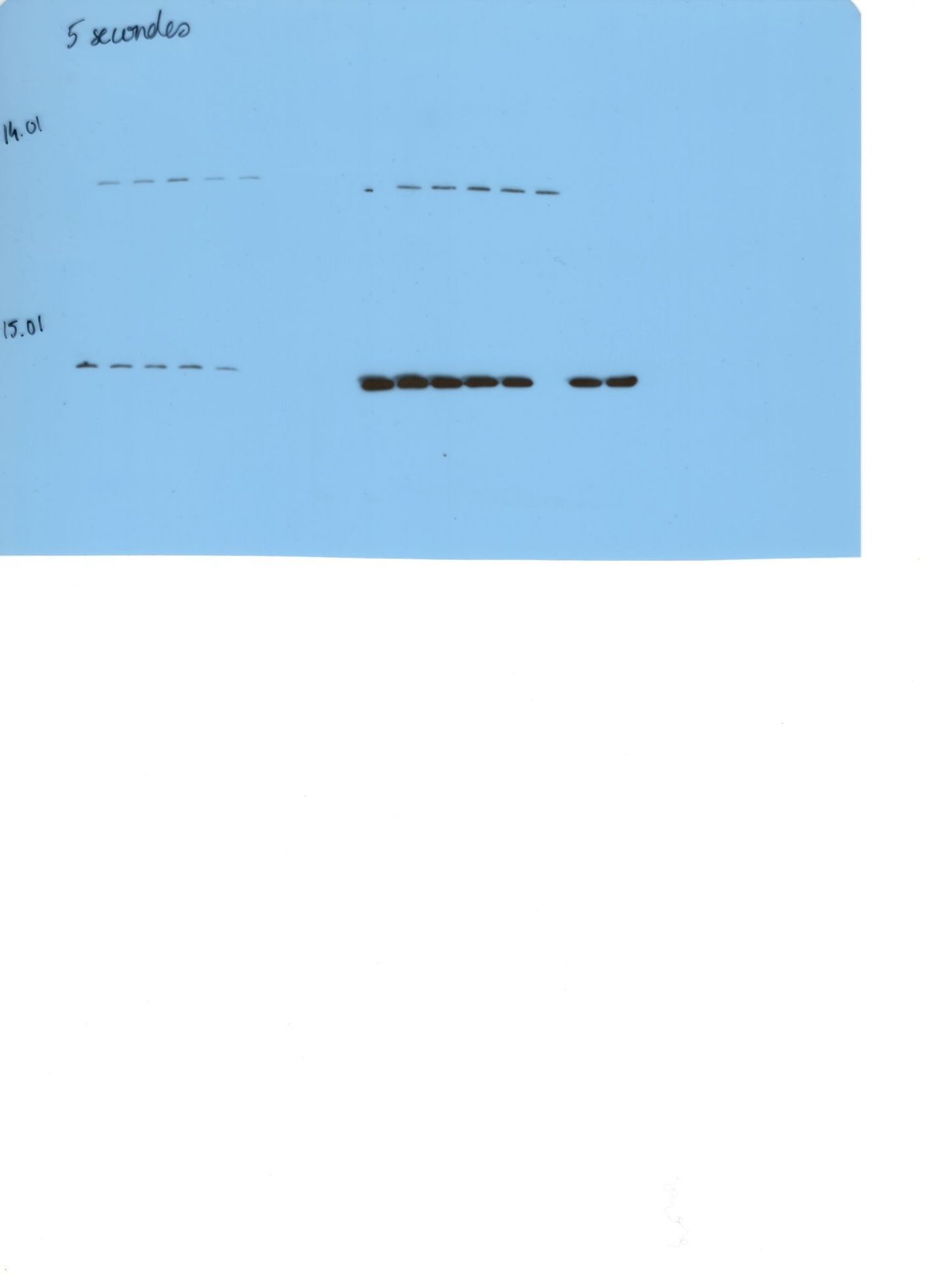


17

Anti-ARF6

17

ARF6 total

**Fig. 1D**


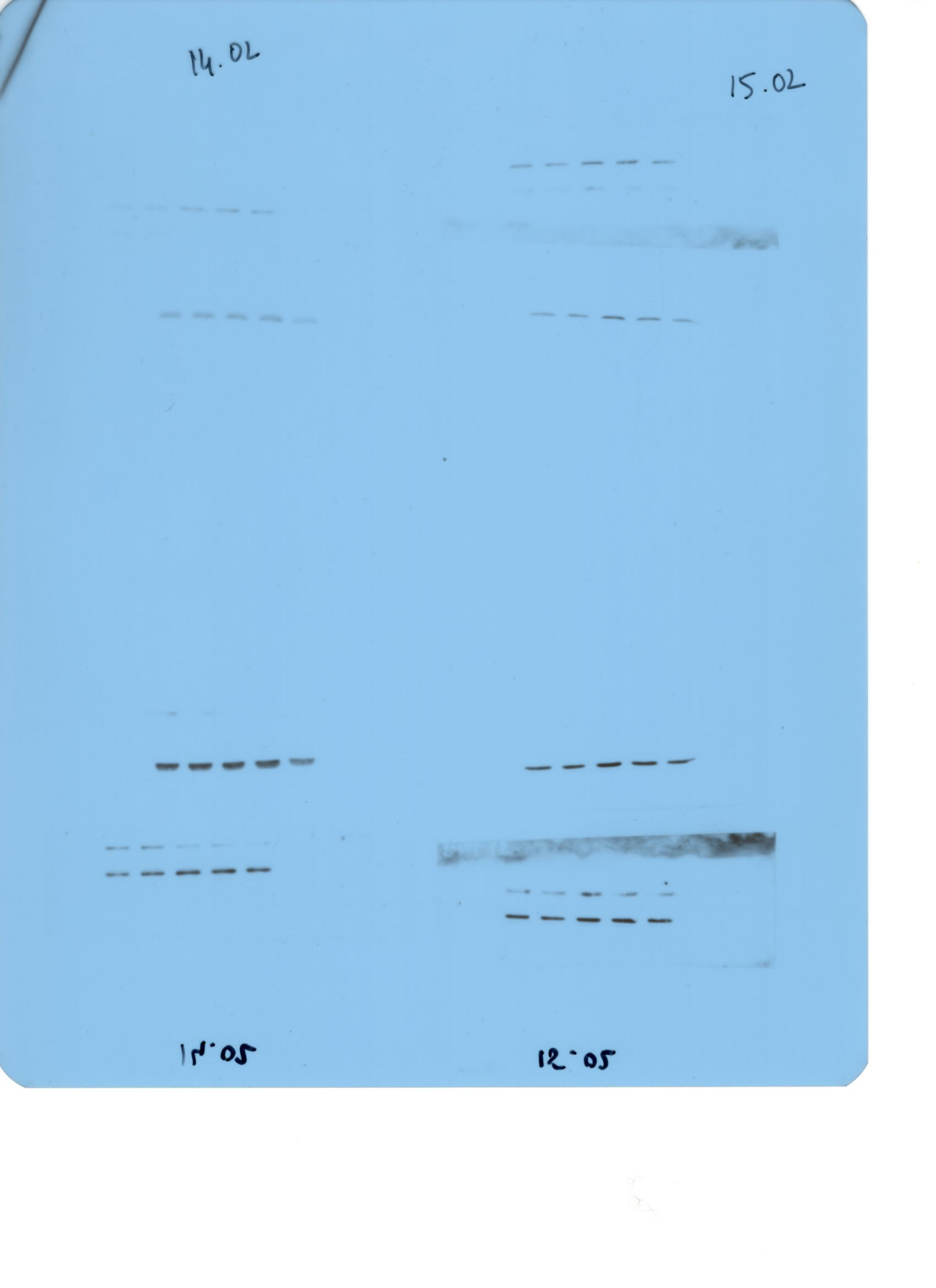

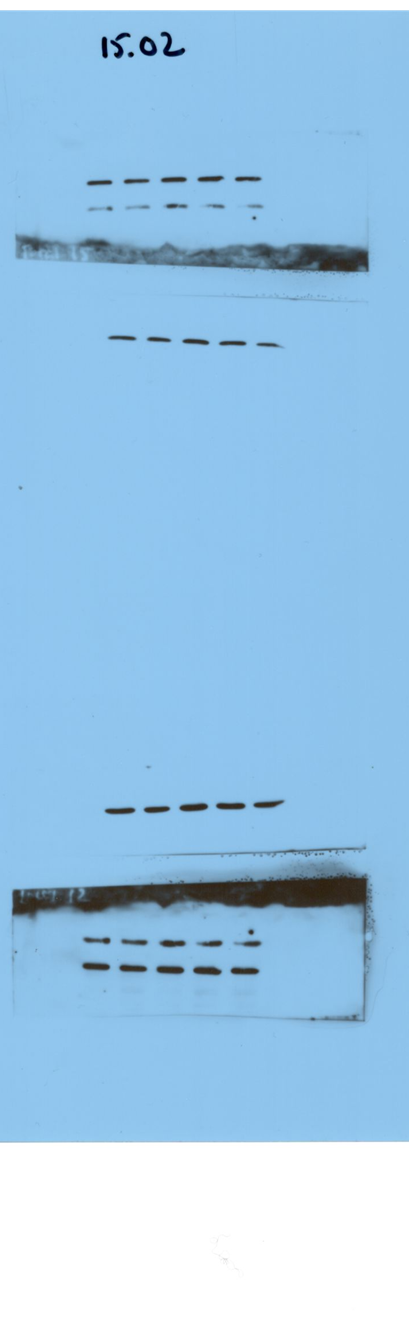


27

27

27

17

27

17

17

Anti-ARF6

(ARF6-GTP)

Anti-ARF6

(ARF6 total)

17

**Fig. 4B**
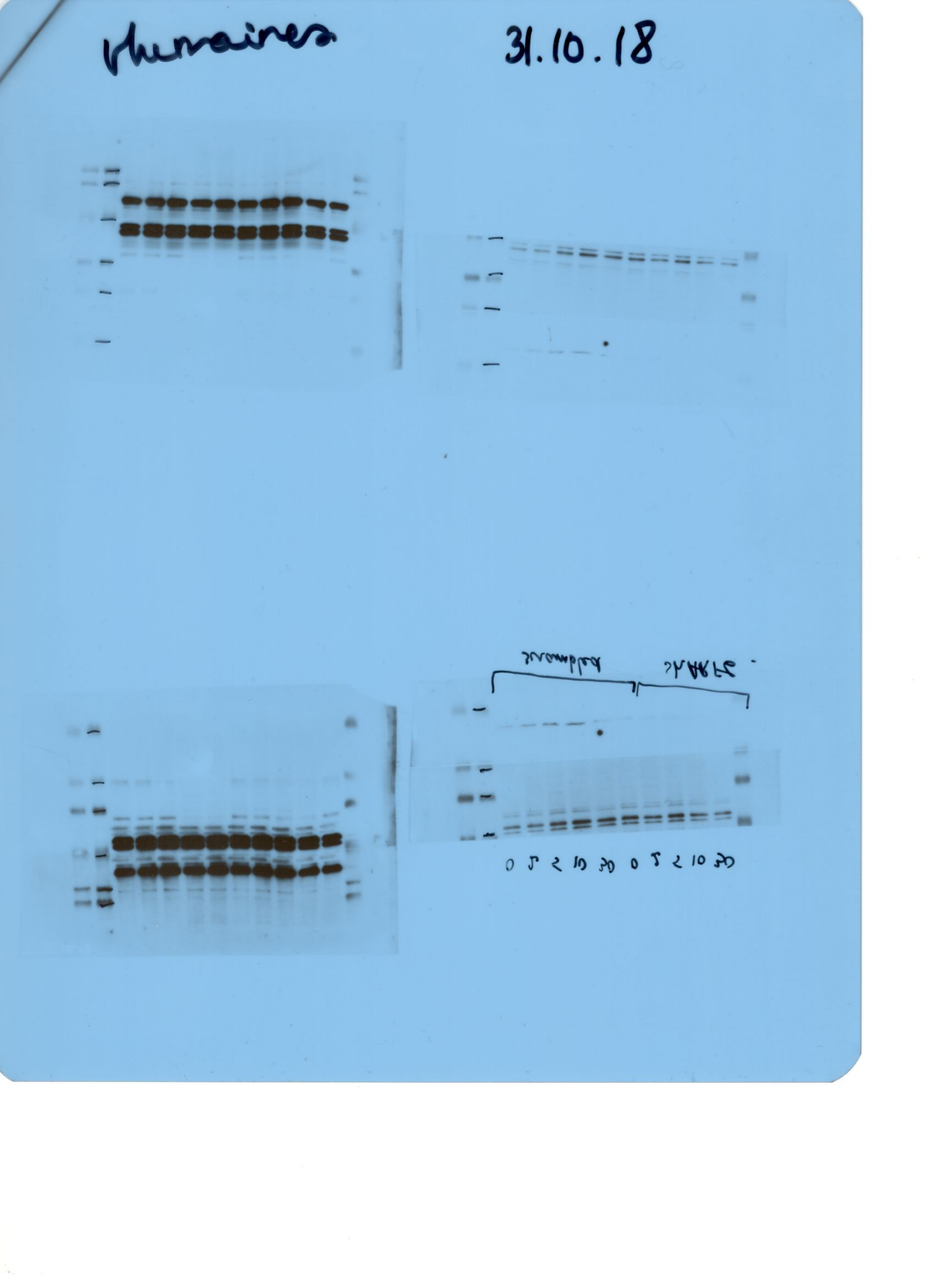


47

33

27

Anti-pERK1/2

17


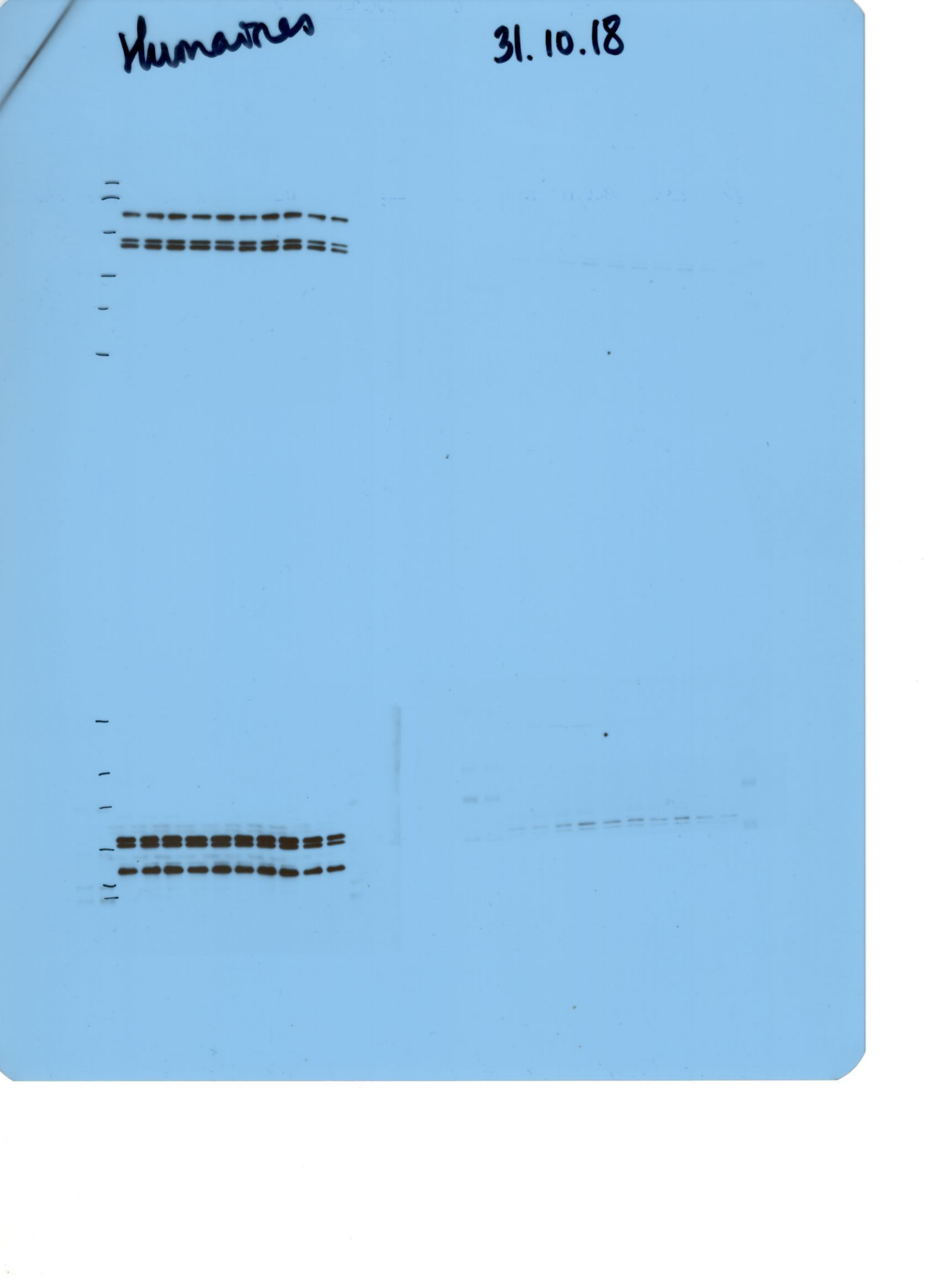


104

82

33

47

Anti-ERK1/2 total

27

17


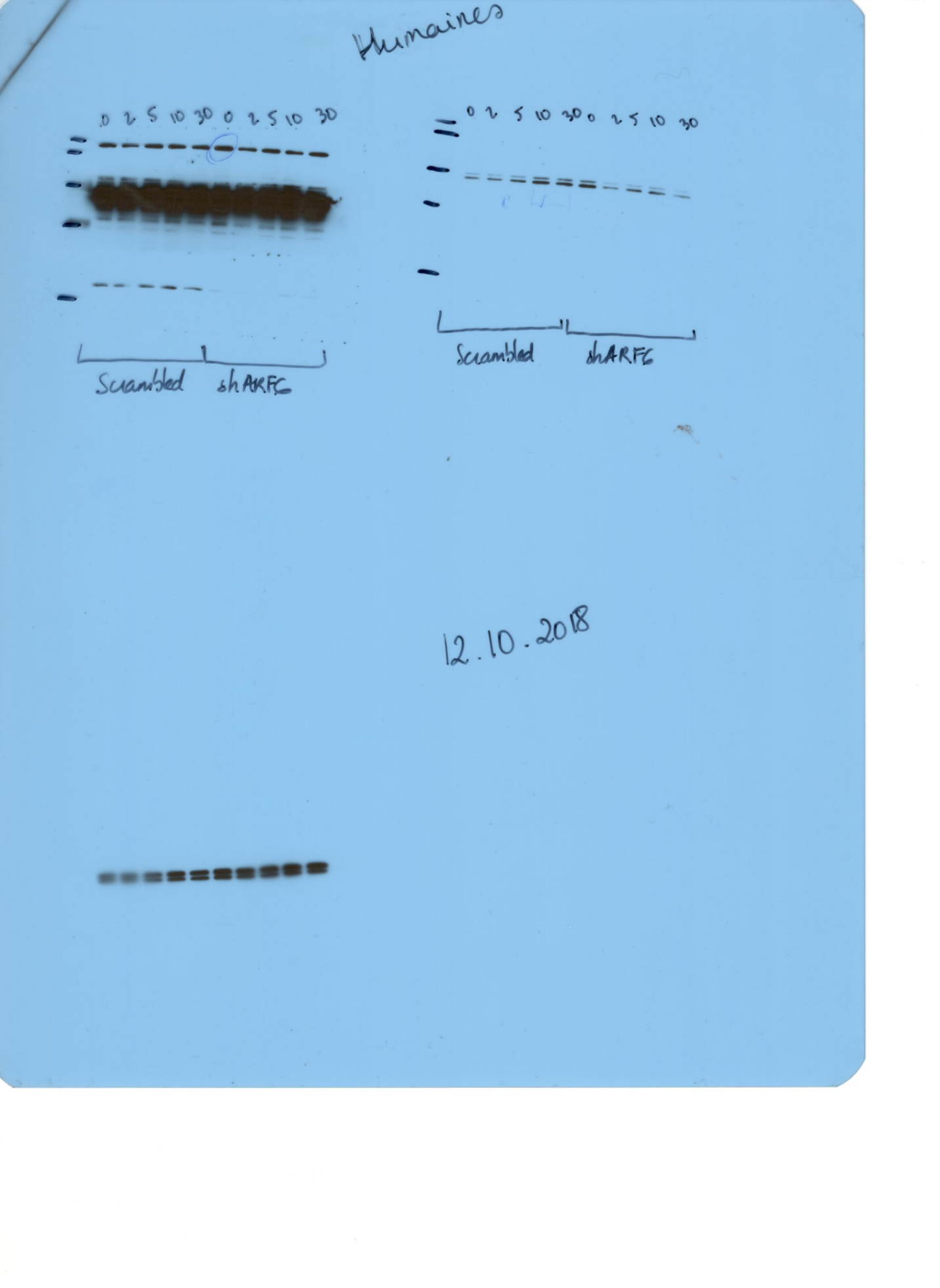


Anti-ARF6

104

47

82

33

17

**Fig. 4D**


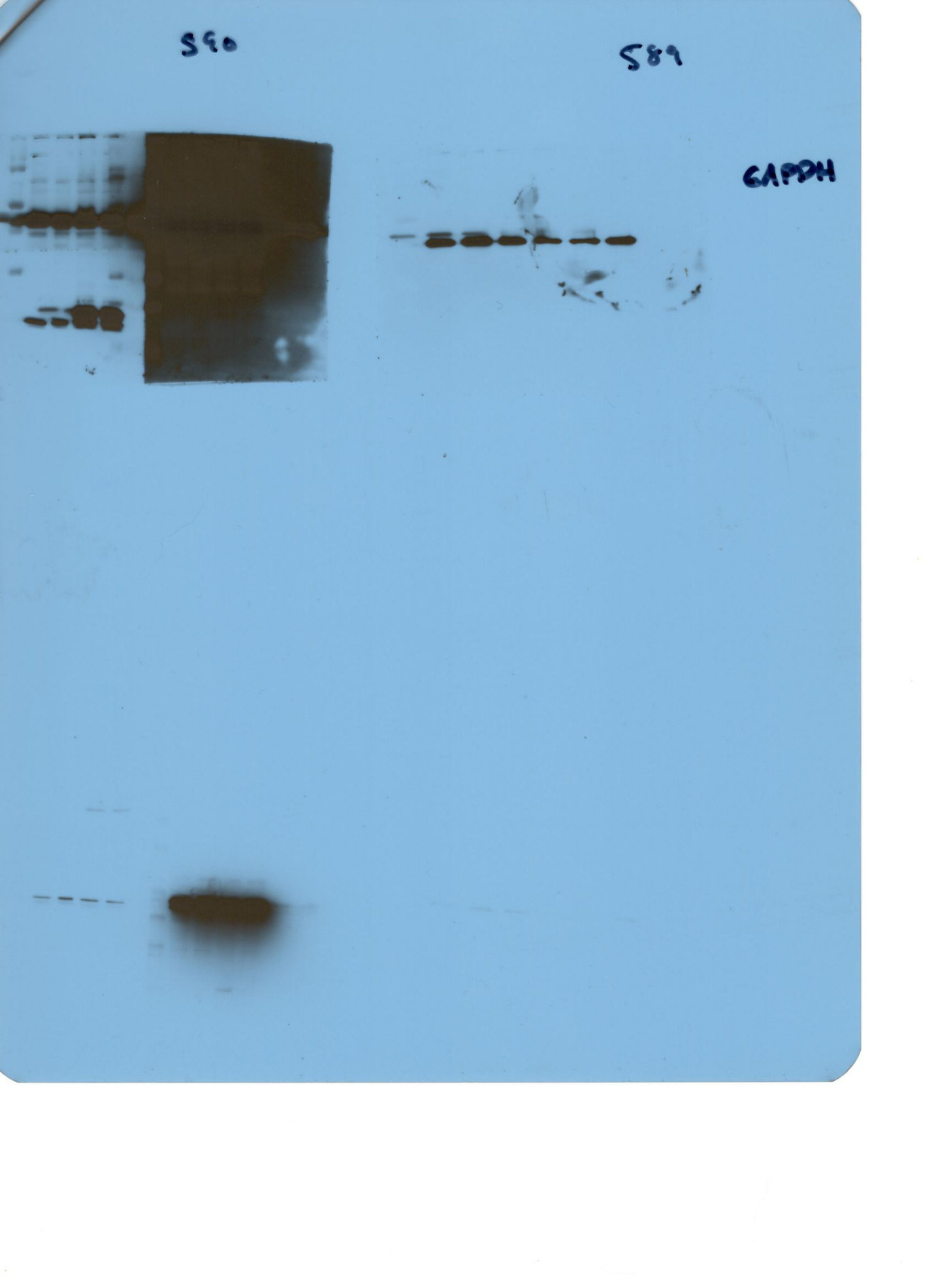

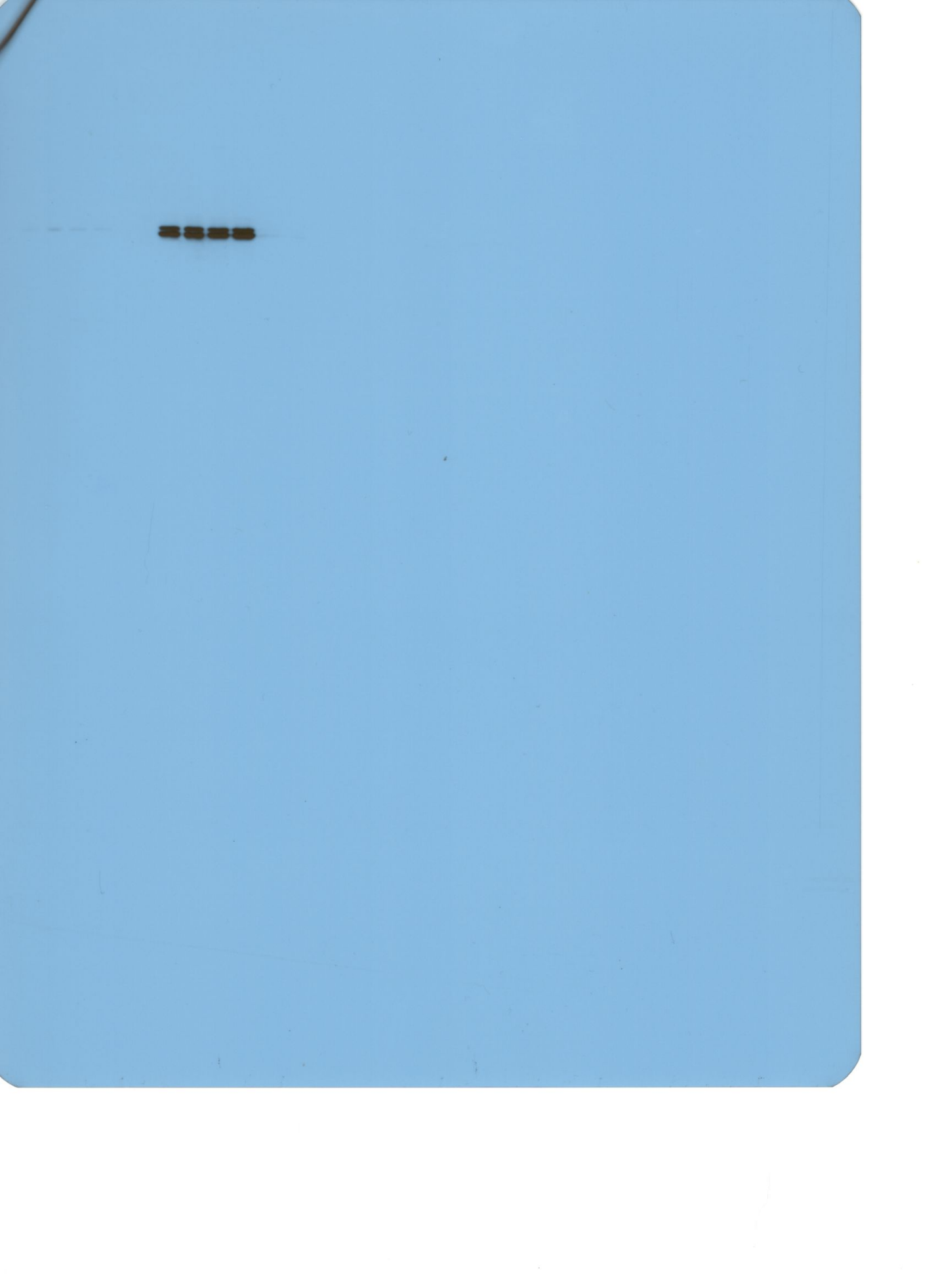


37

25

20

75

50

Anti-ERK1/2

20

Anti-pERK1/2

75

50

37

25

Exposure time 30 sec touch


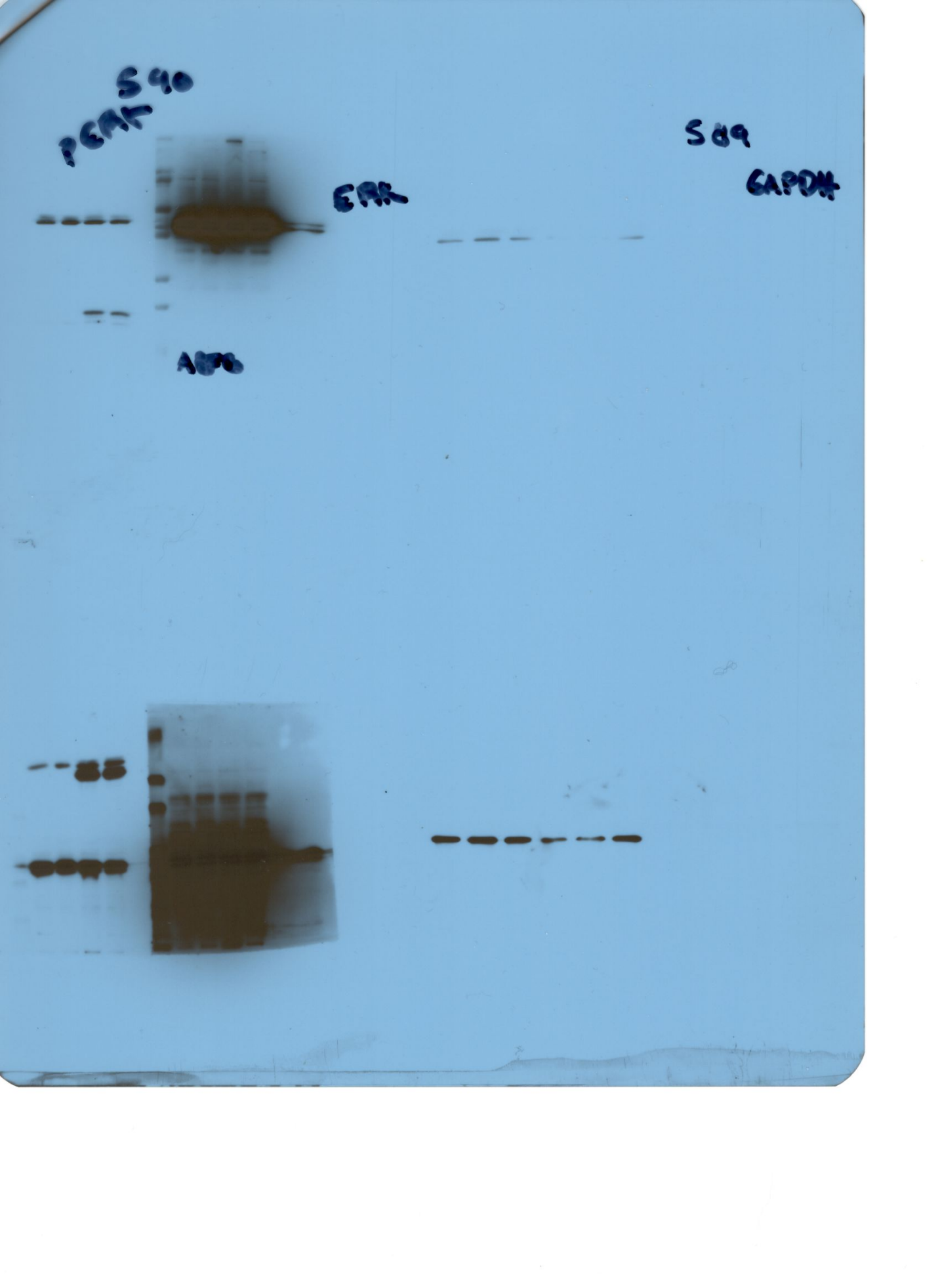


15

20

37

50

25

Anti-ARF6

**Fig. 5B**


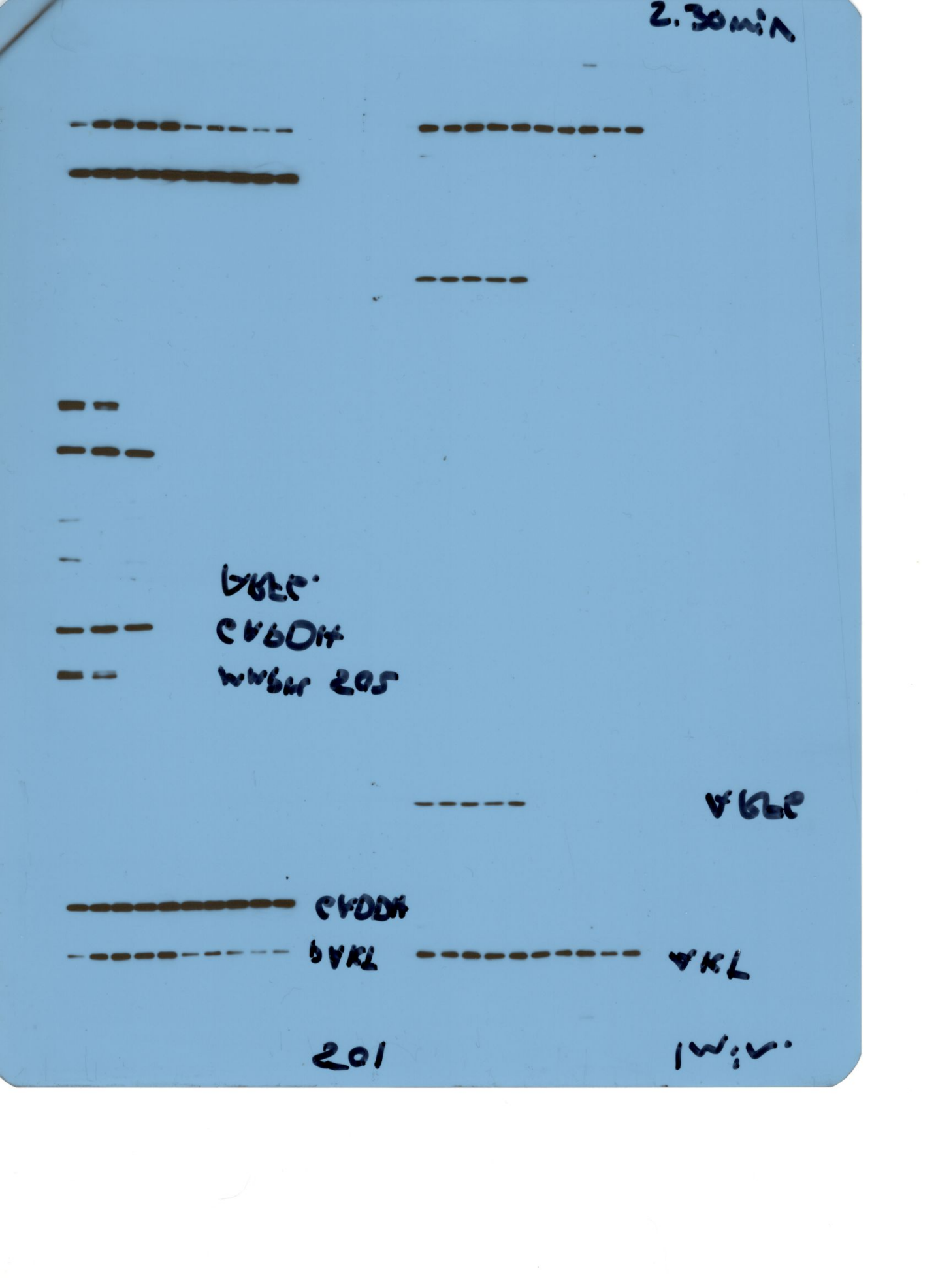


Anti-AKT

82

47


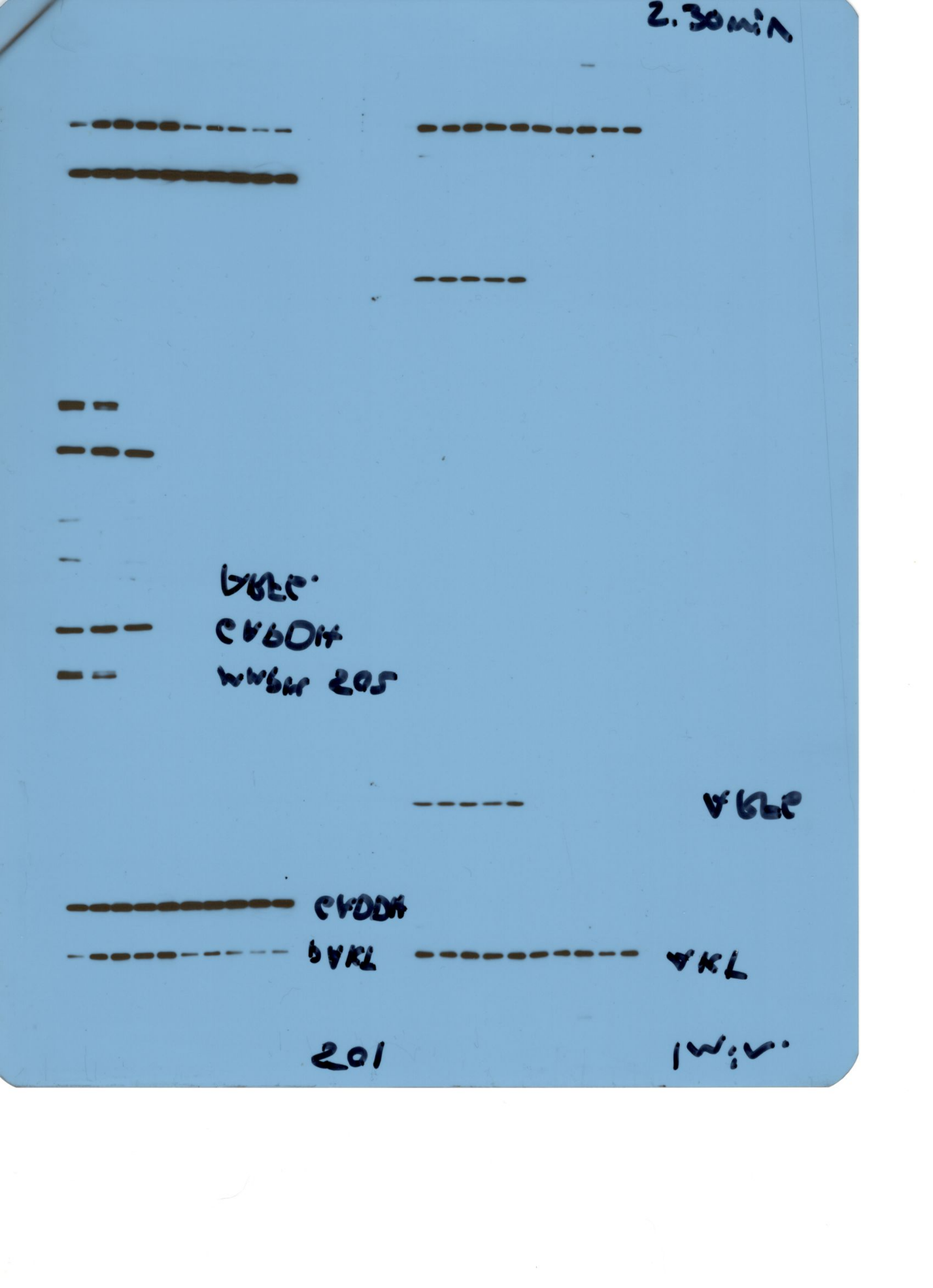


50

75

37


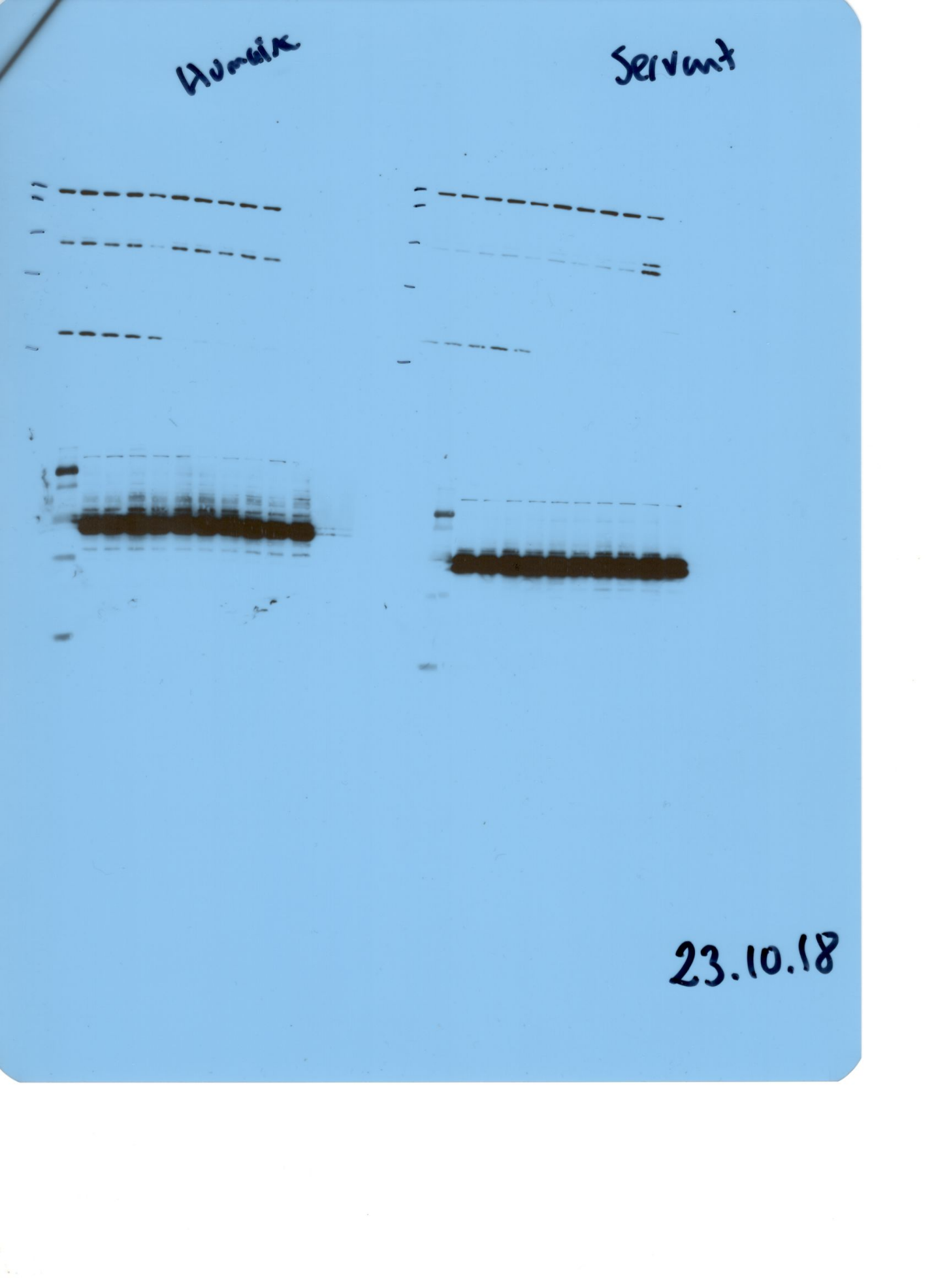


Anti-ARF6

17

104

33

82

47

**Fig. 6B**

**
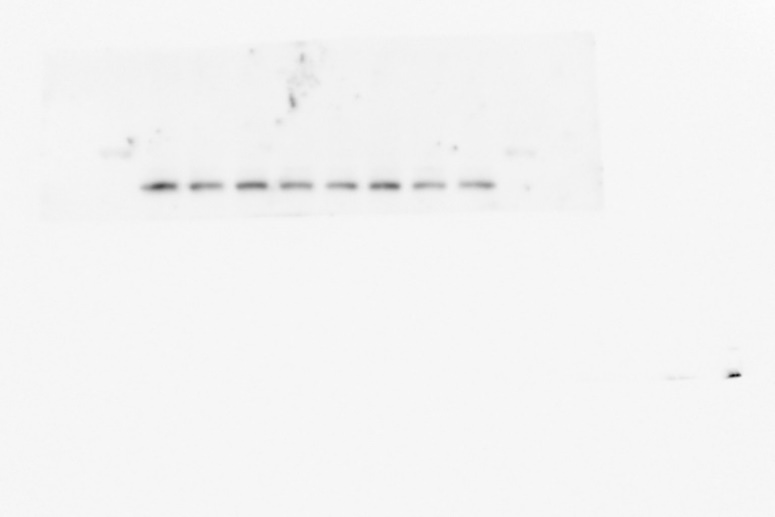
**

50

75

Anti-PAK1/2





50

75

Anti-pPAK1/2





25

20

Anti-ARF6

**Fig. 6D**


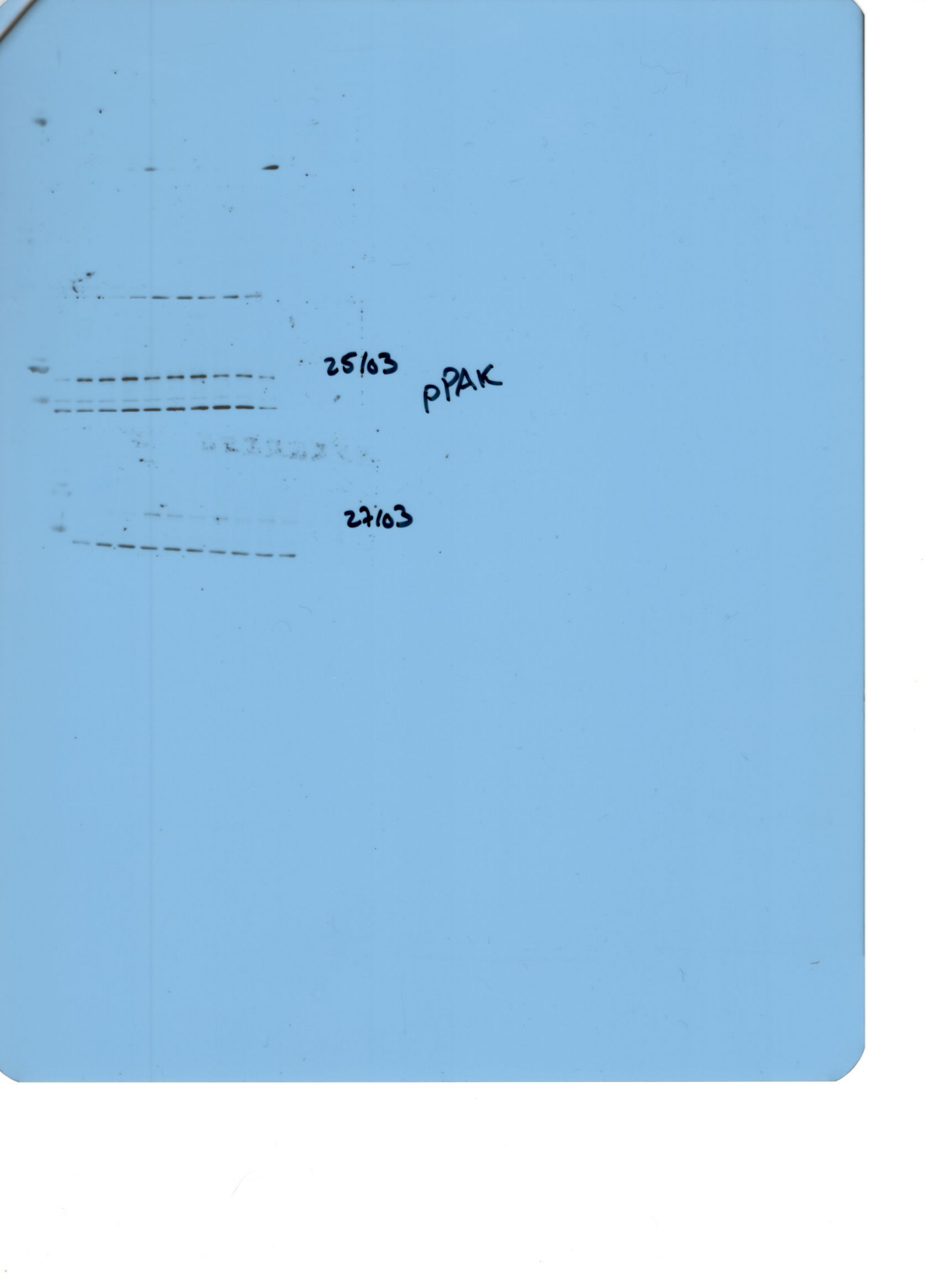


50

75


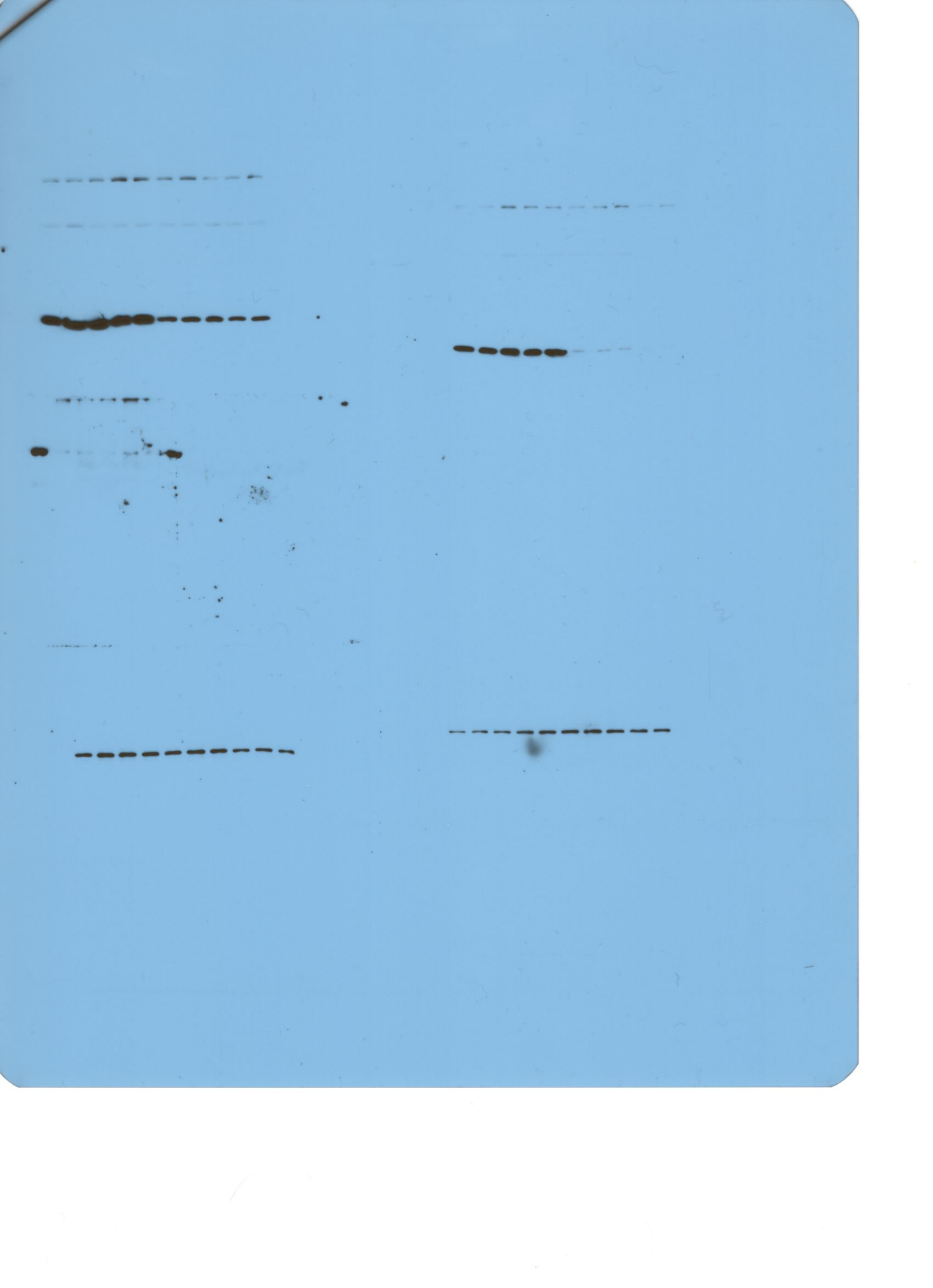


50

75

Anti-PAK1/2


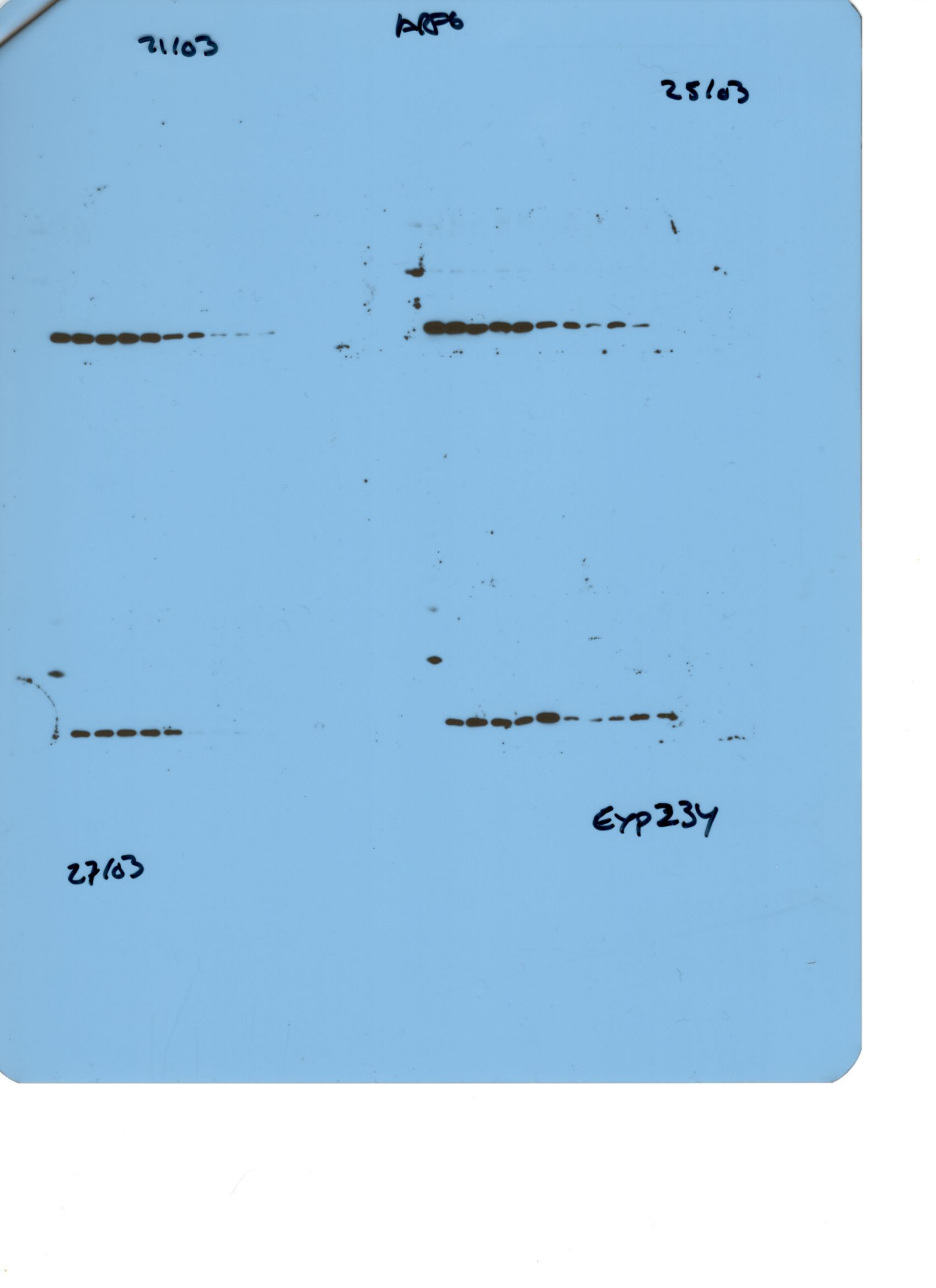


20

Anti-ARF6

25

**Fig.7A**

Anti-MMP2





75

50

Anti-Actin





37

50

Anti-ARF6





25

20

**Fig. 7E**


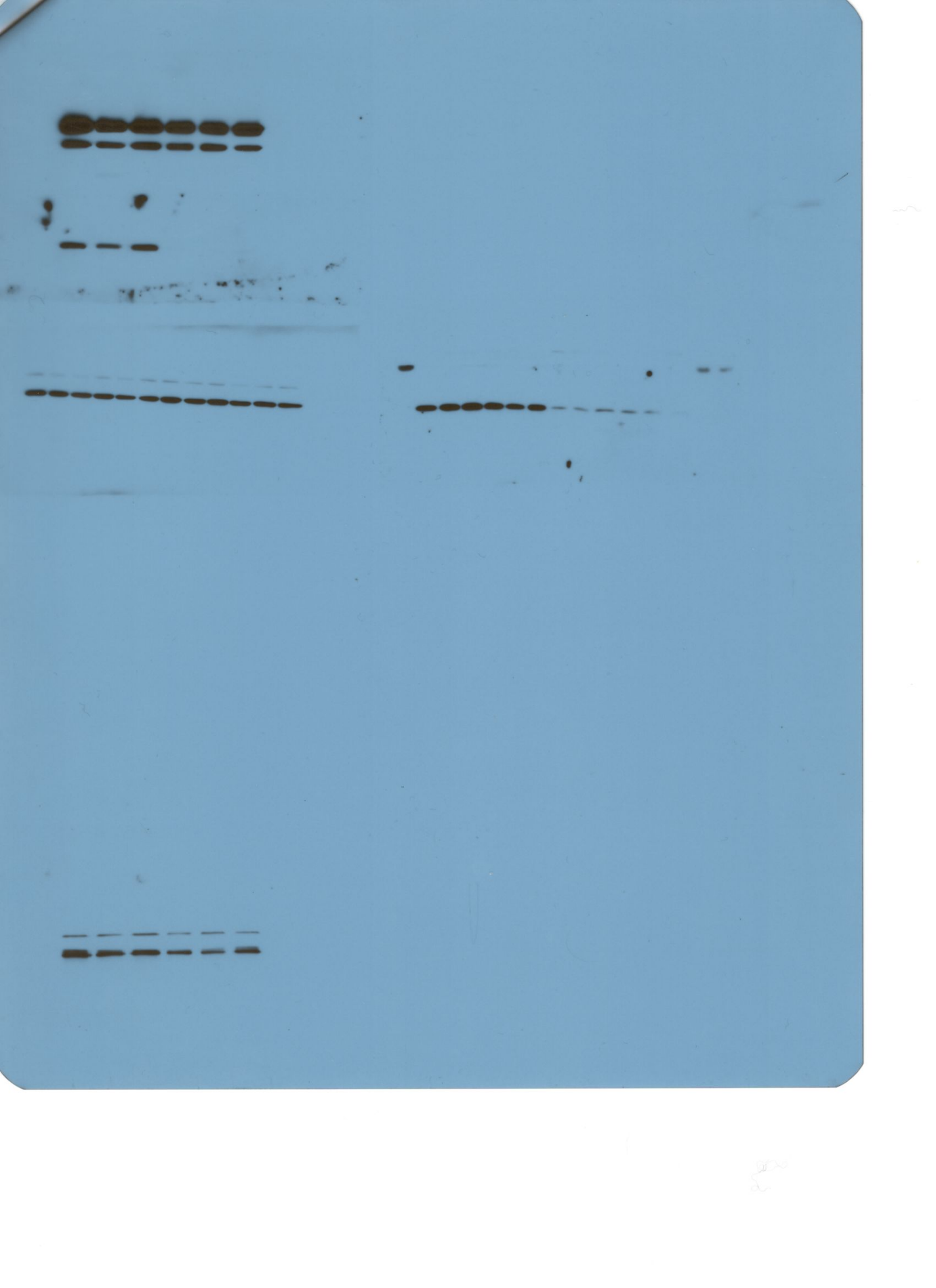


25

20

75

50

Anti-ARF6

Anti-Actin

Anti-MMP14


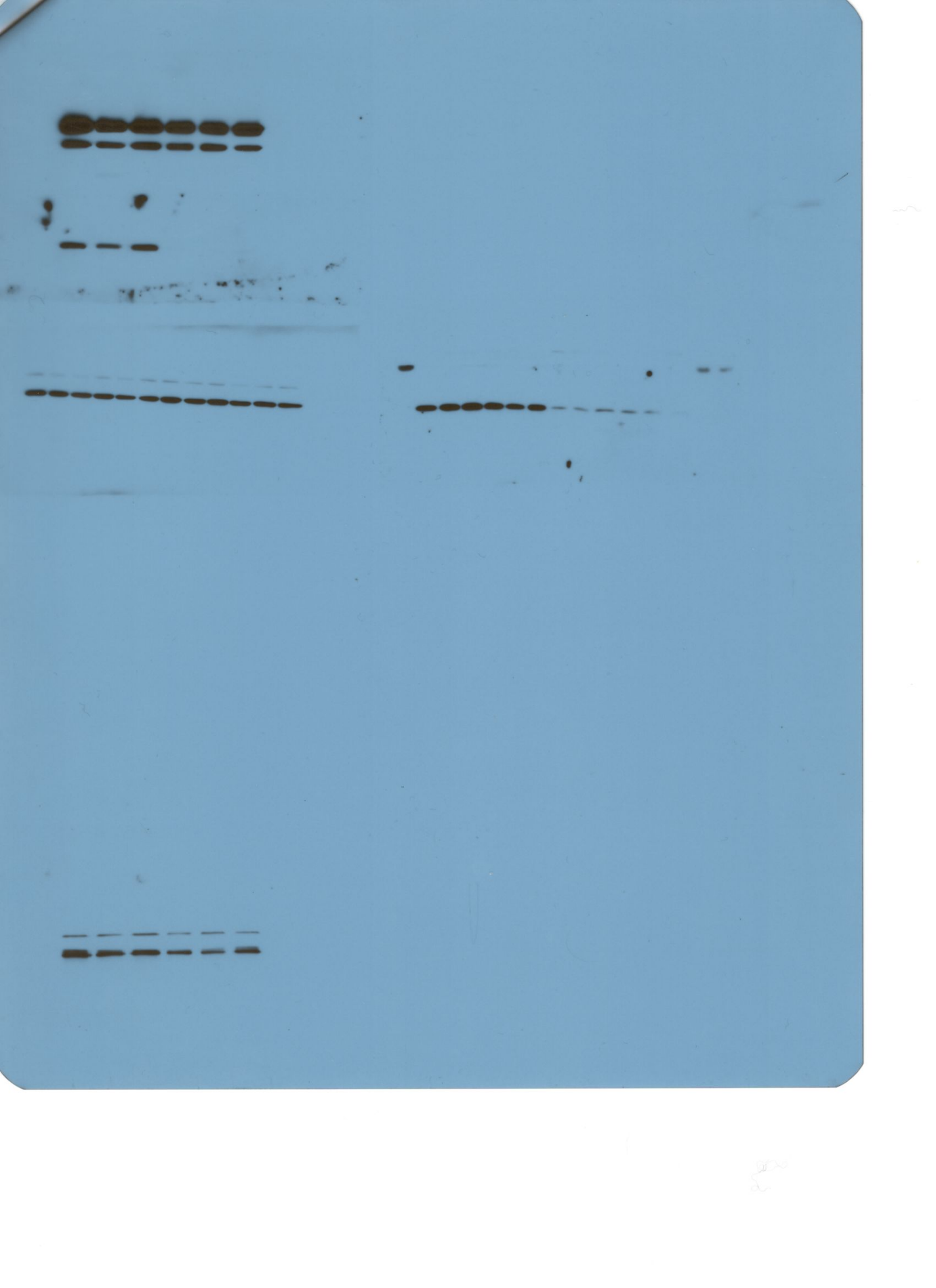


75

50

**Fig. 7F**


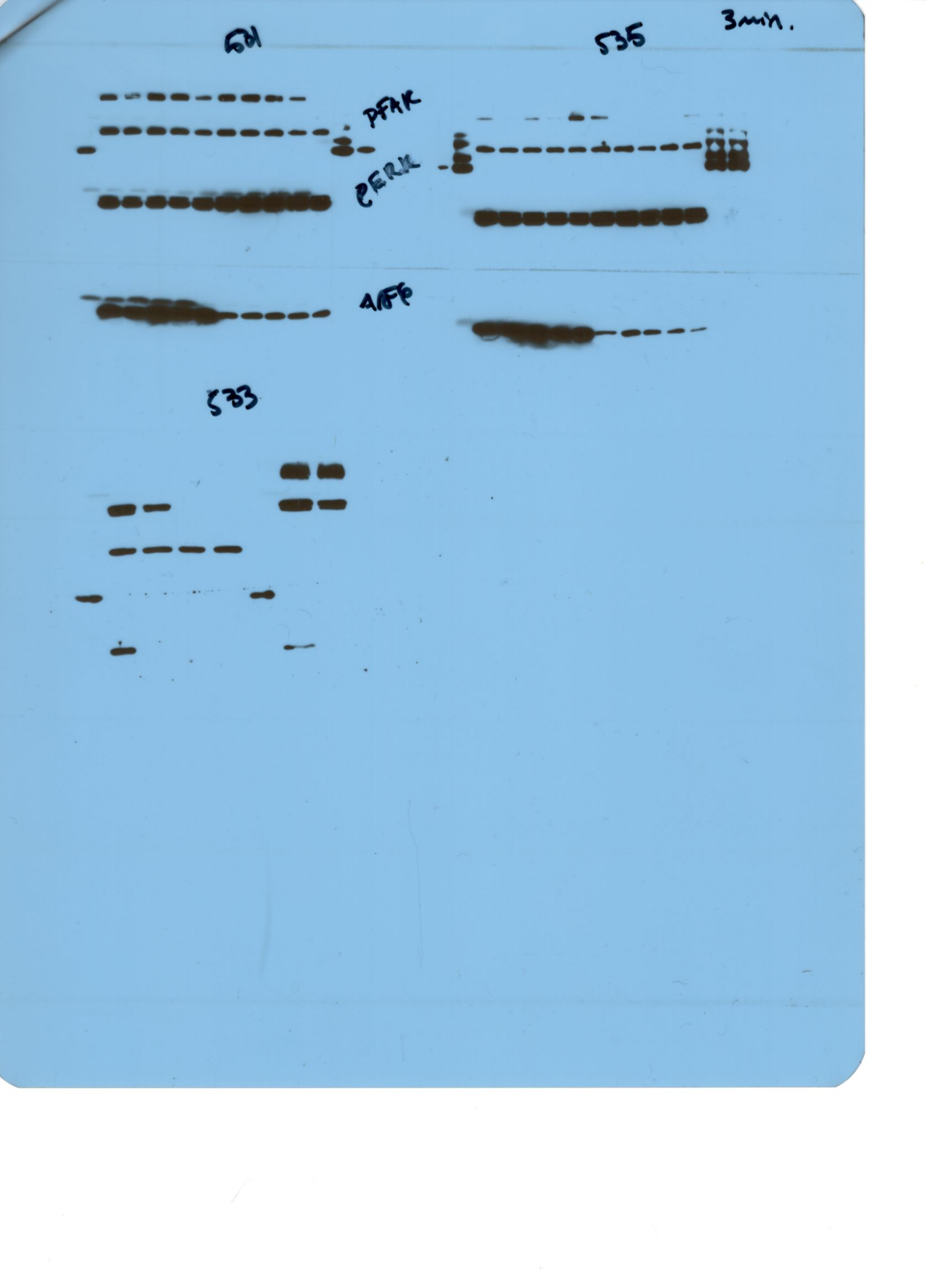


20

50

75

25

Anti-ARF6

Anti-βCatenin


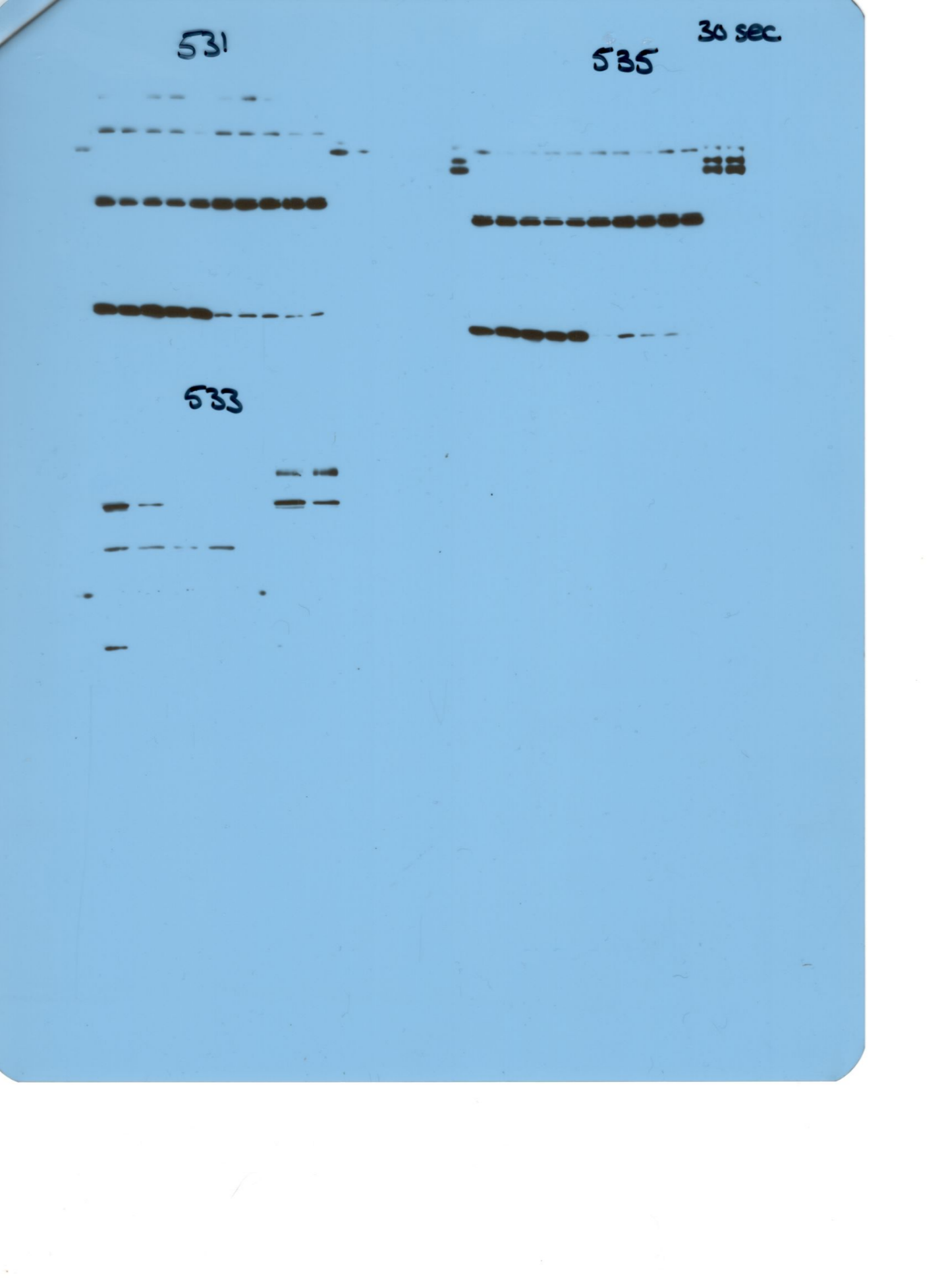


20

50

75

25

Anti-MMP14

**Fig. 7G**





25

20

Anti-ARF6




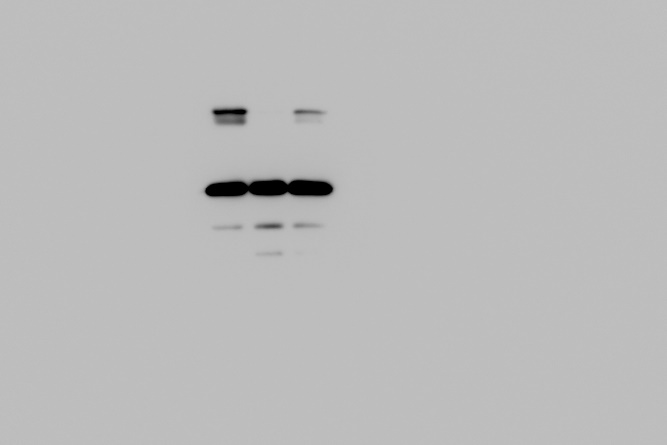


Anti-MMP14

Anti-GAPDH

25

37

50

75

37

50

75

***Antibody Specificity***


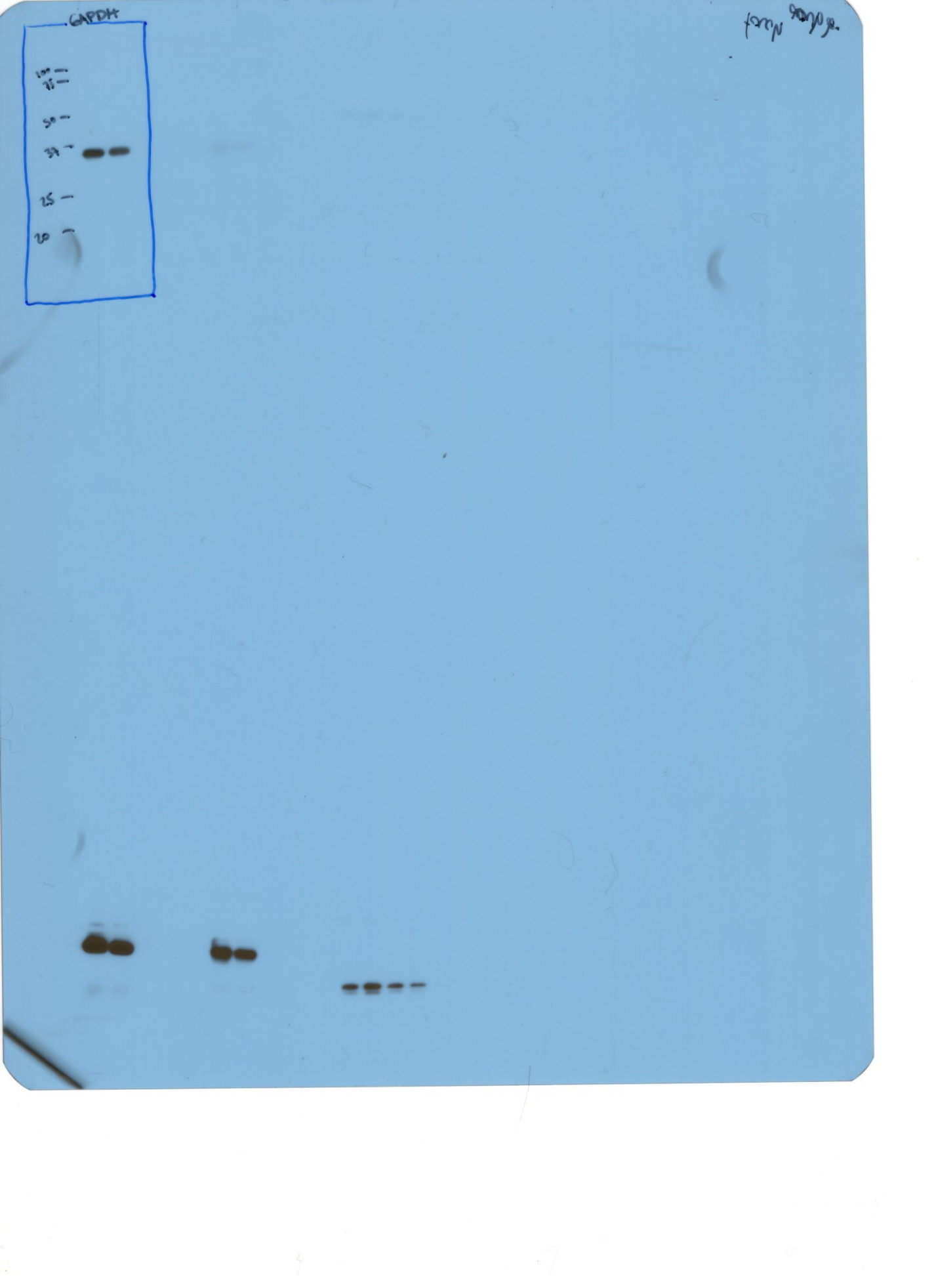

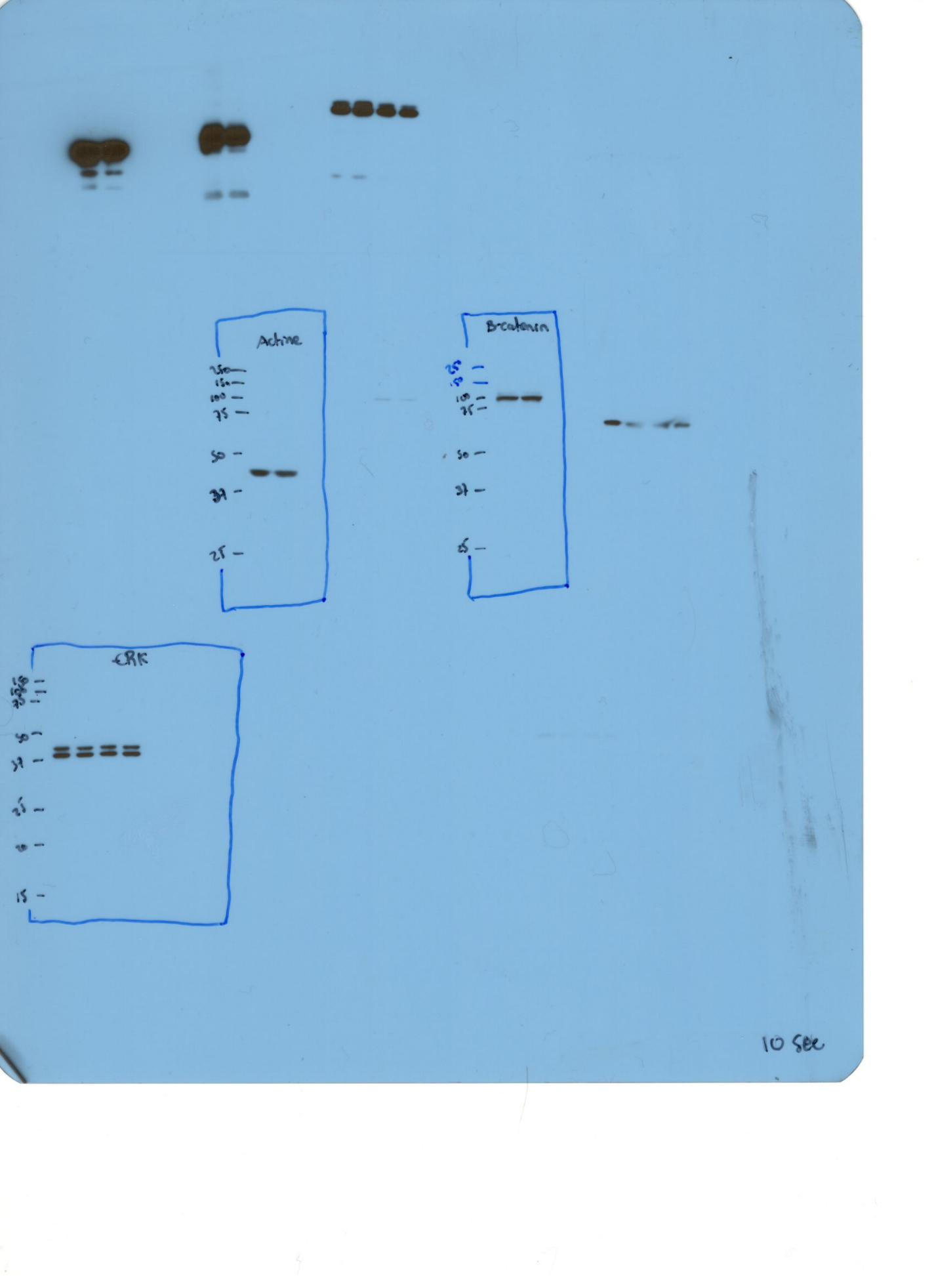

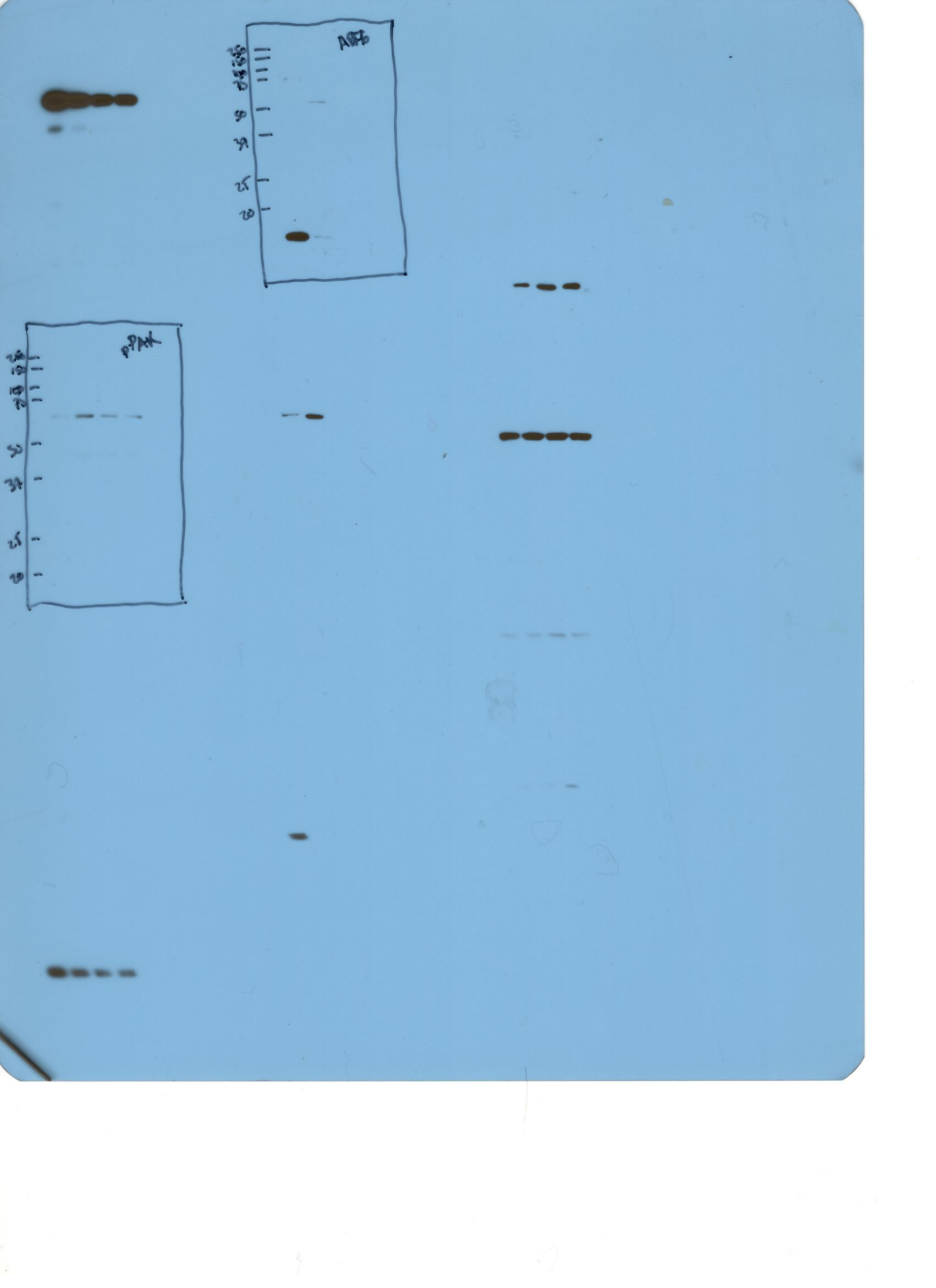

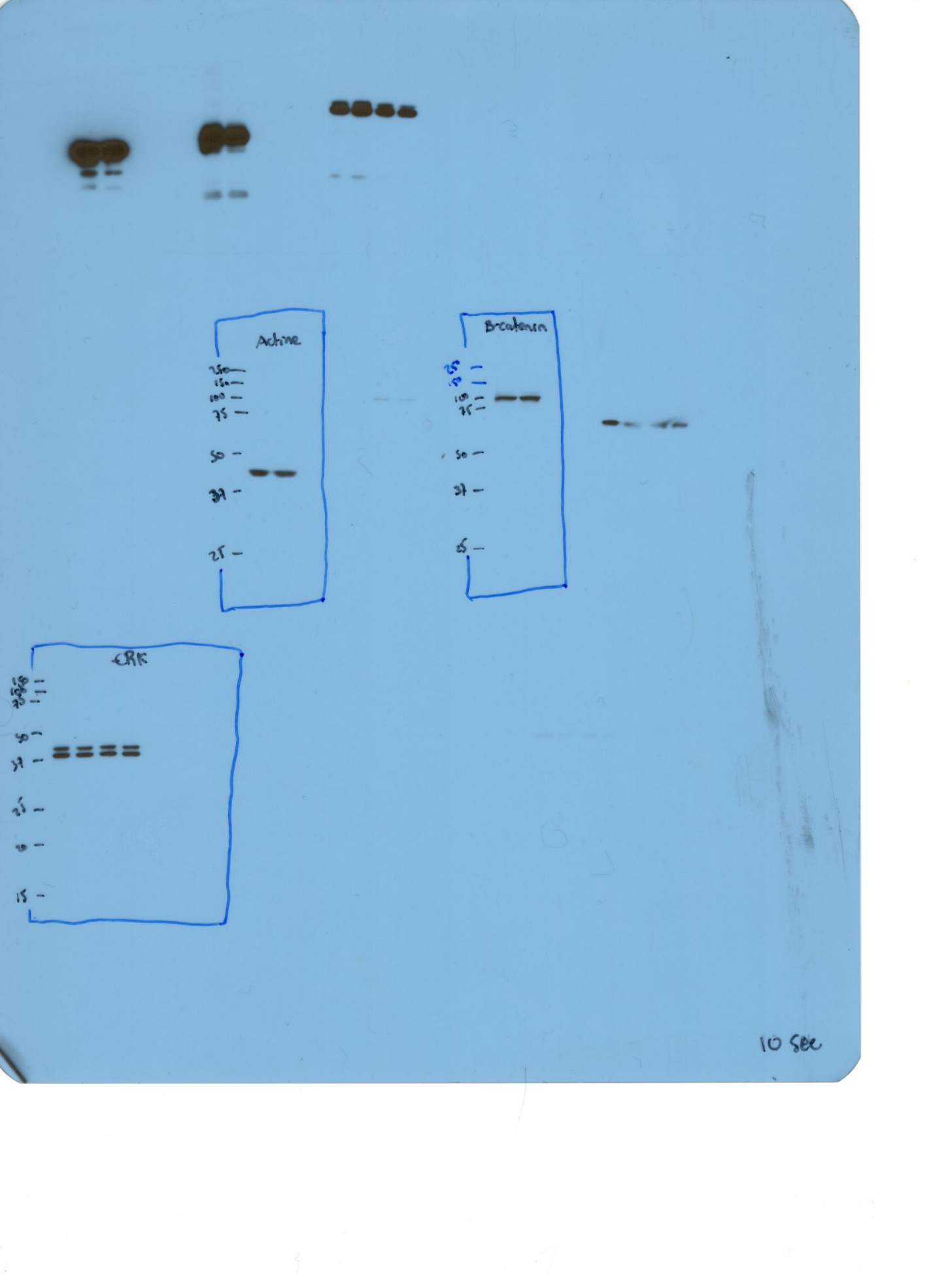

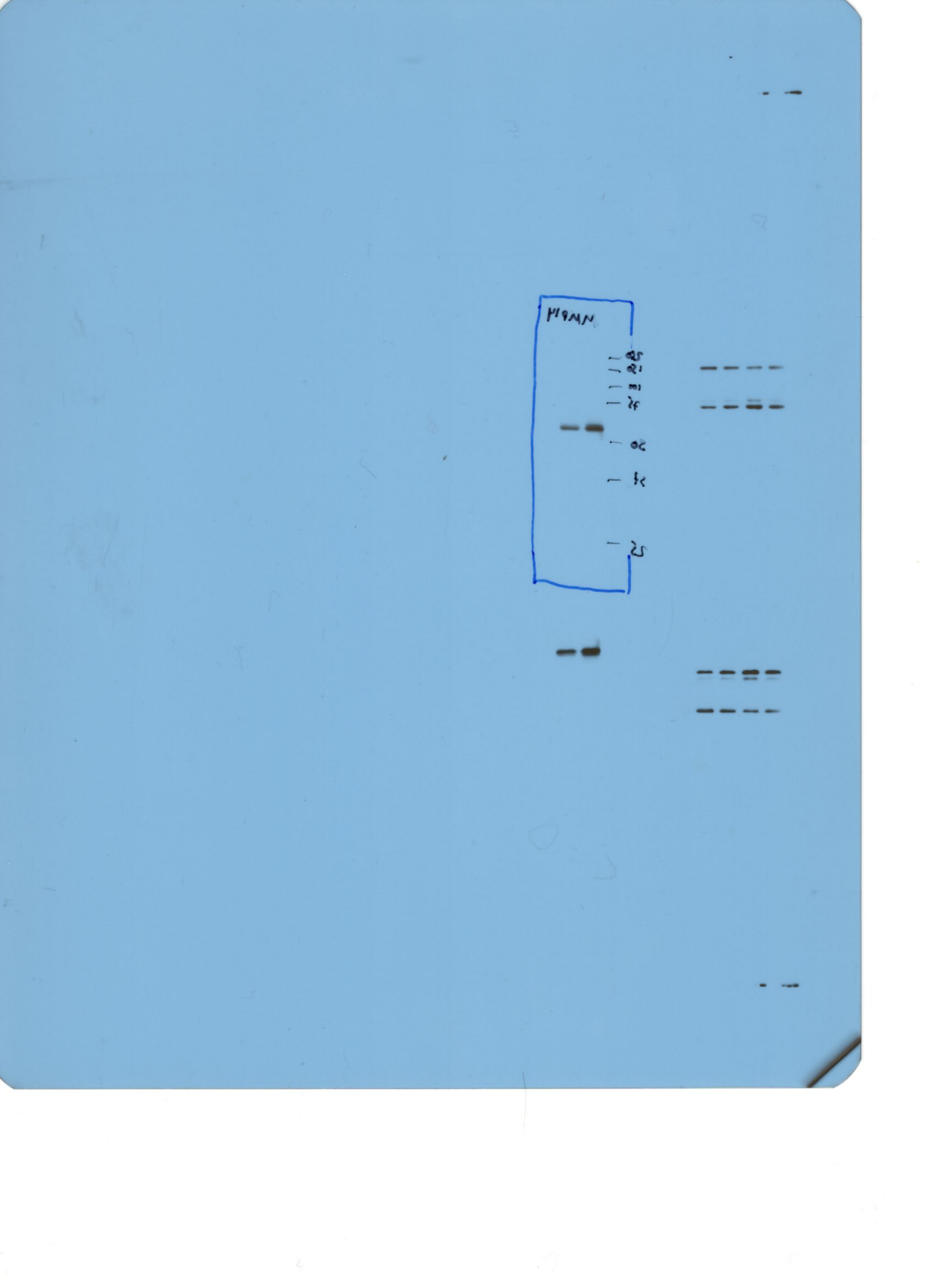


1 2

1 2

1 2

1 2

1 2

Lane 1 : shRNA Ctl whole cell lysate

Lane 2 : shRNA ARF6 whole cell lysate

MMP2


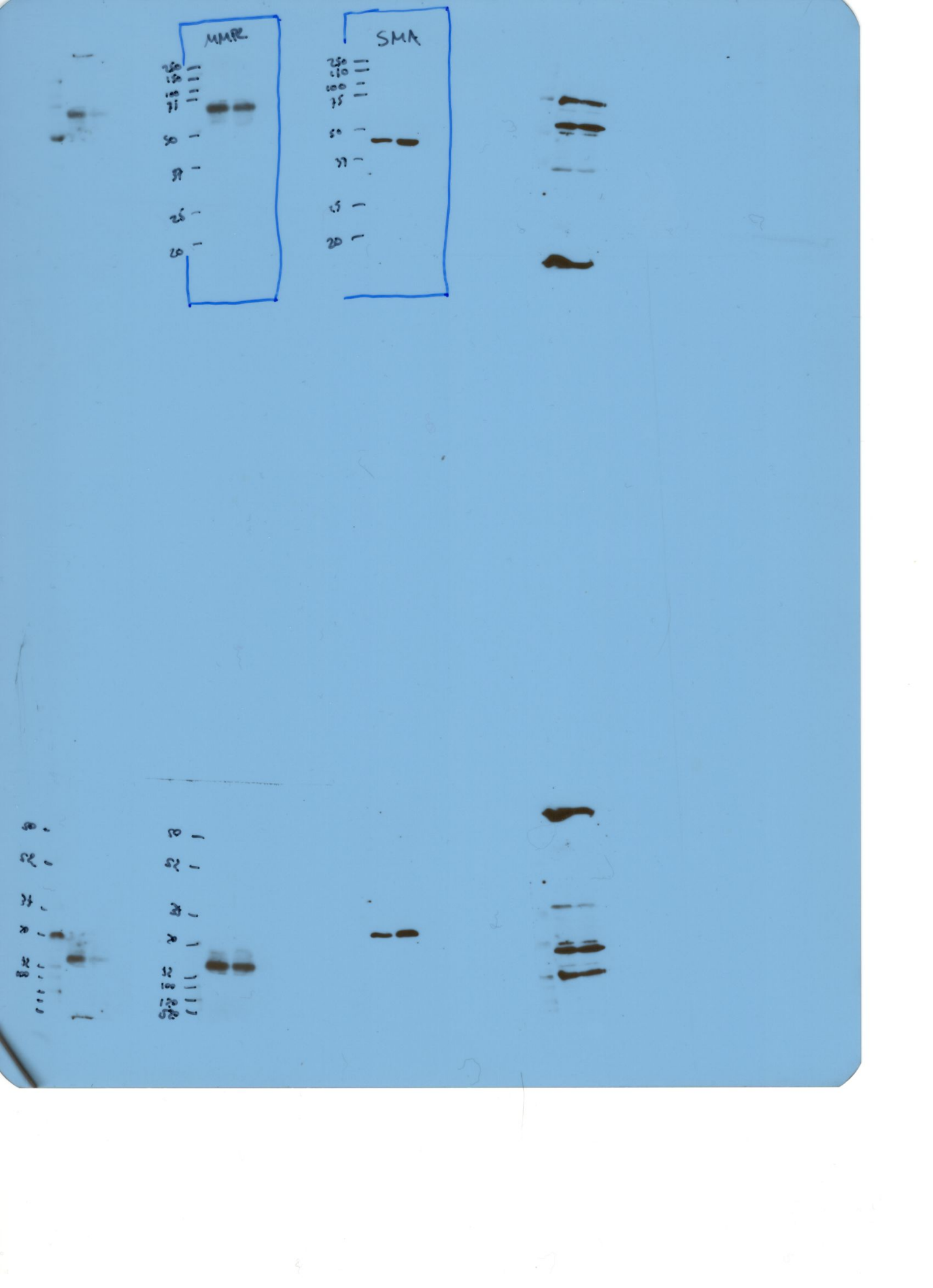


Lane 1 : shRNA Ctl supernatant

1 2

Lane 2 : shRNA ARF6 supernatant


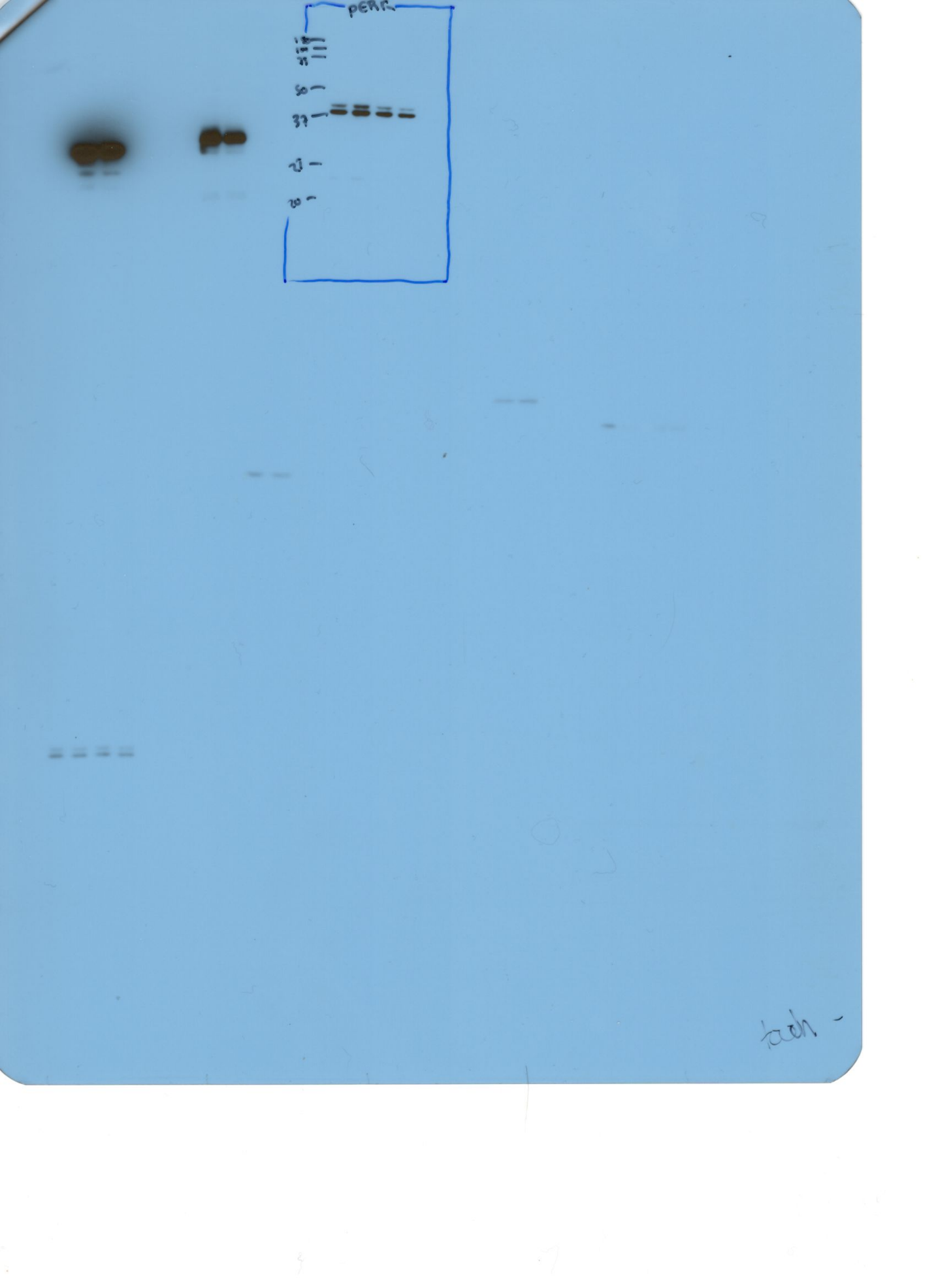

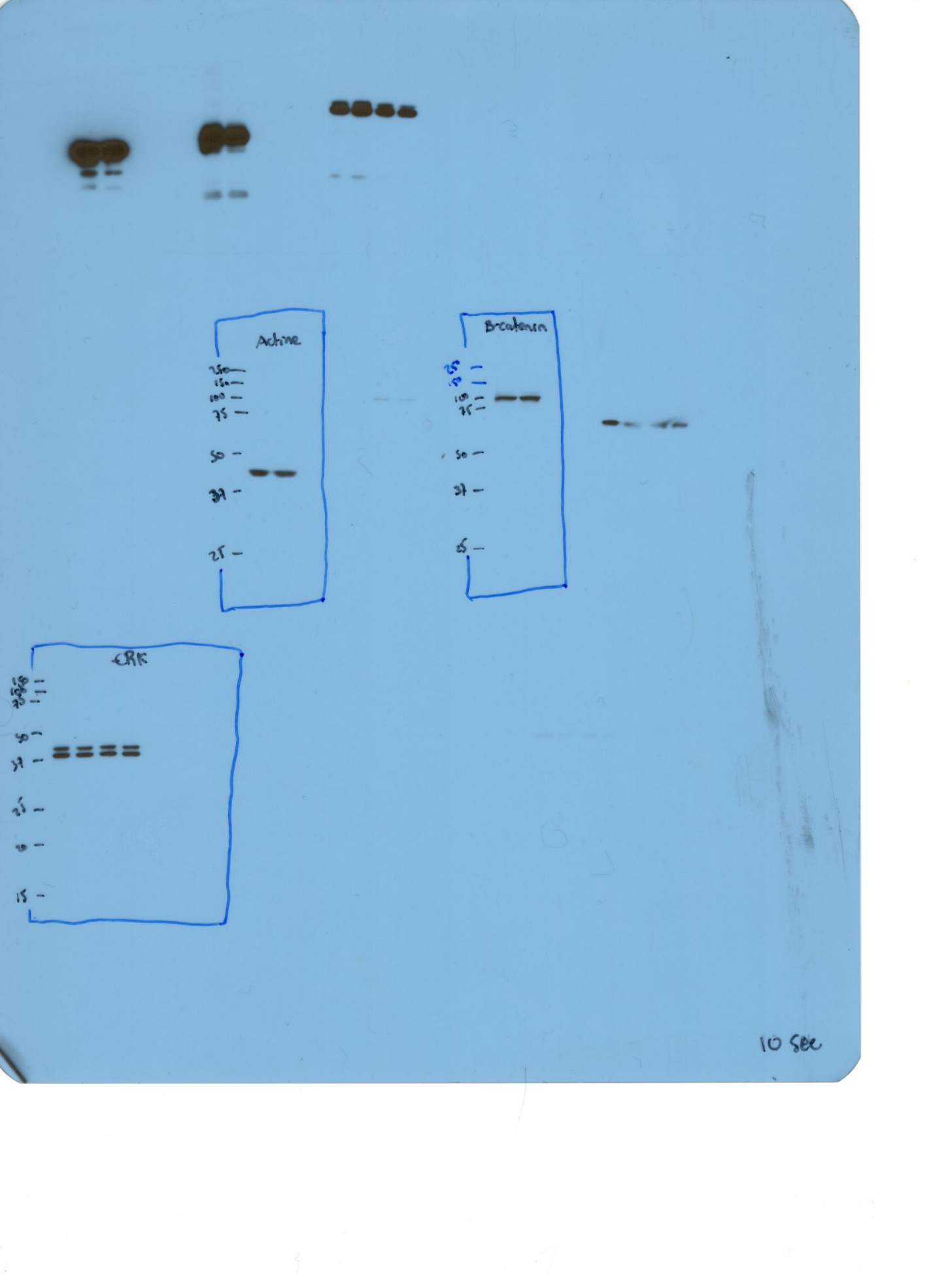


p-ERK1/2

ERK1/2

1 2 3 4

1 2 3 4

Lane 1 : shRNA Ctl 0 min

Lane 2 : shRNA Ctl 30 min

Lane 3 : shRNA Ctl 60 min

Lane 4 : shRNA Ctl 120 min


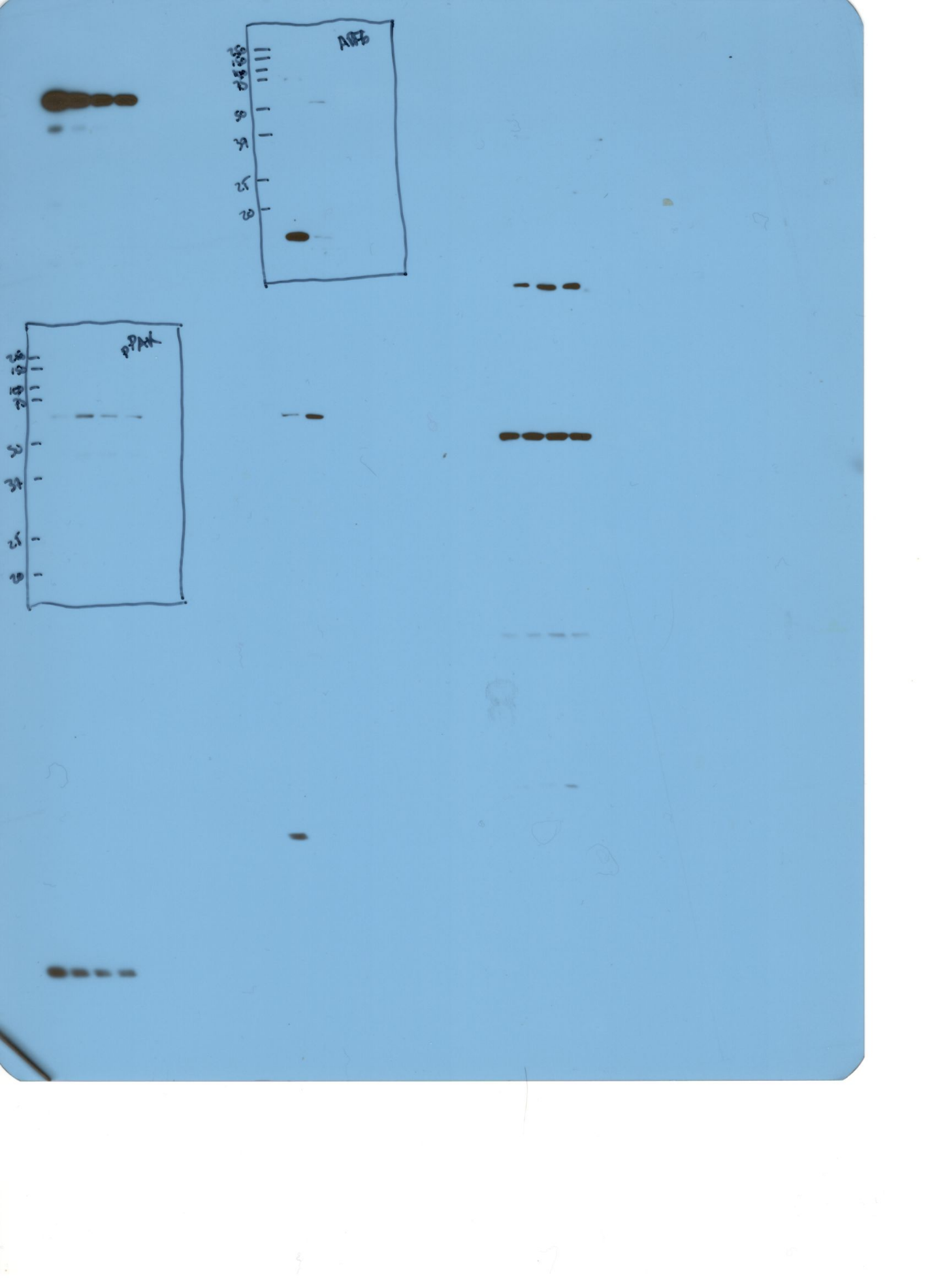

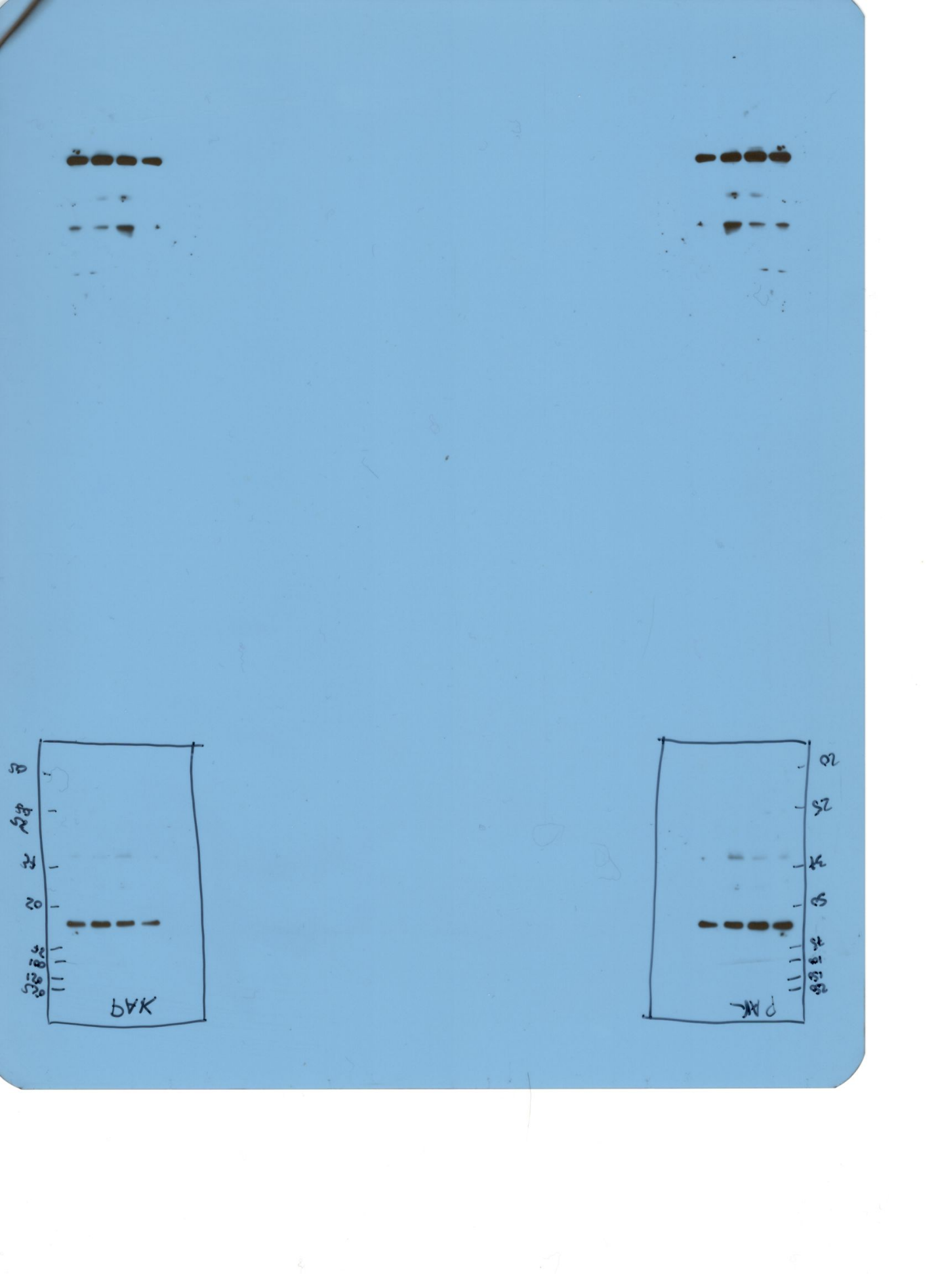


1 2 3 4

PAK1/2

p-PAK1/2

1 2 3 4

Lane 1 : shRNA Ctl 0 min

Lane 2 : shRNA Ctl 30 min

Lane 3 : shRNA Ctl 60 min

Lane 4 : shRNA Ctl 120 min


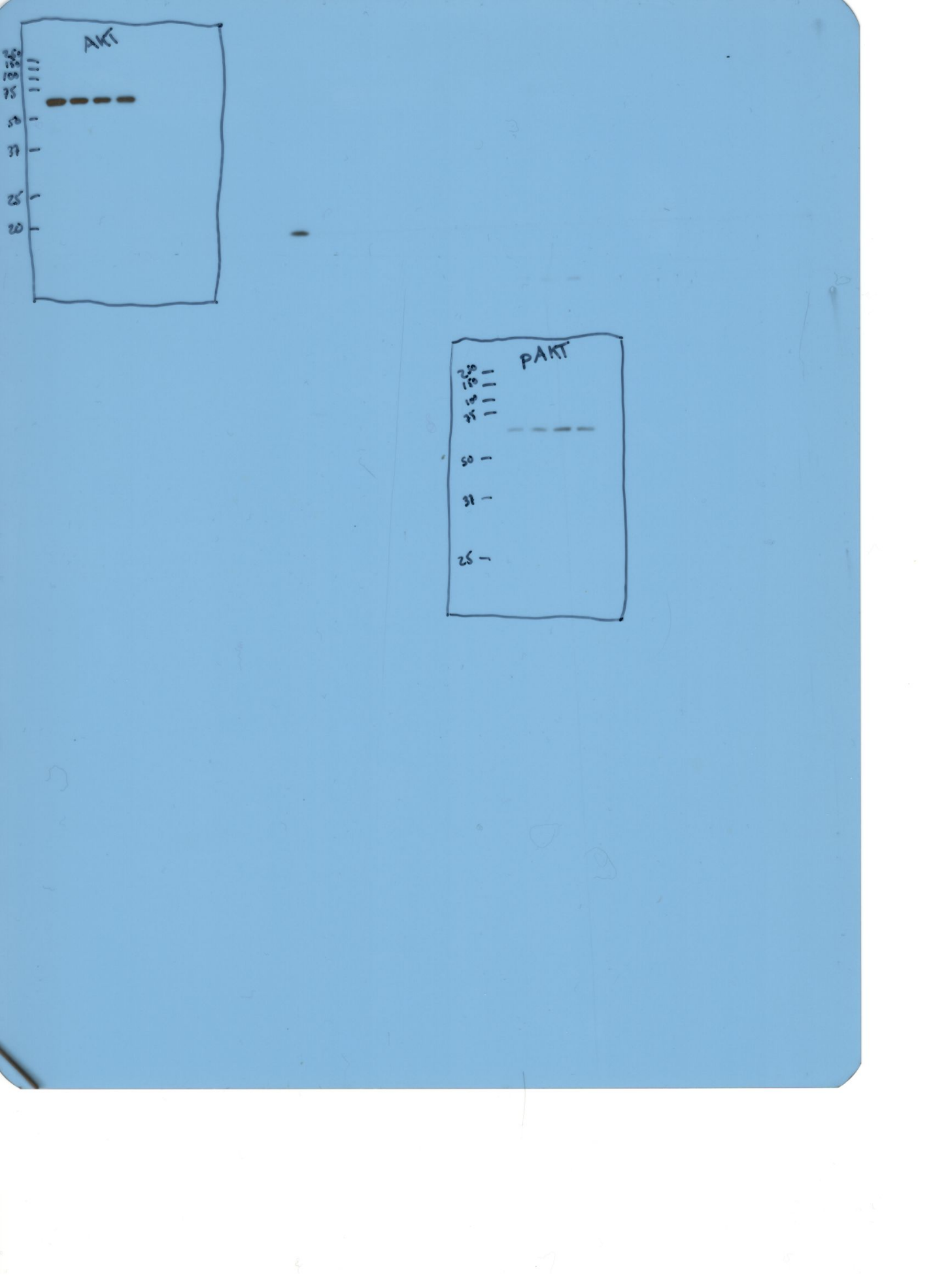

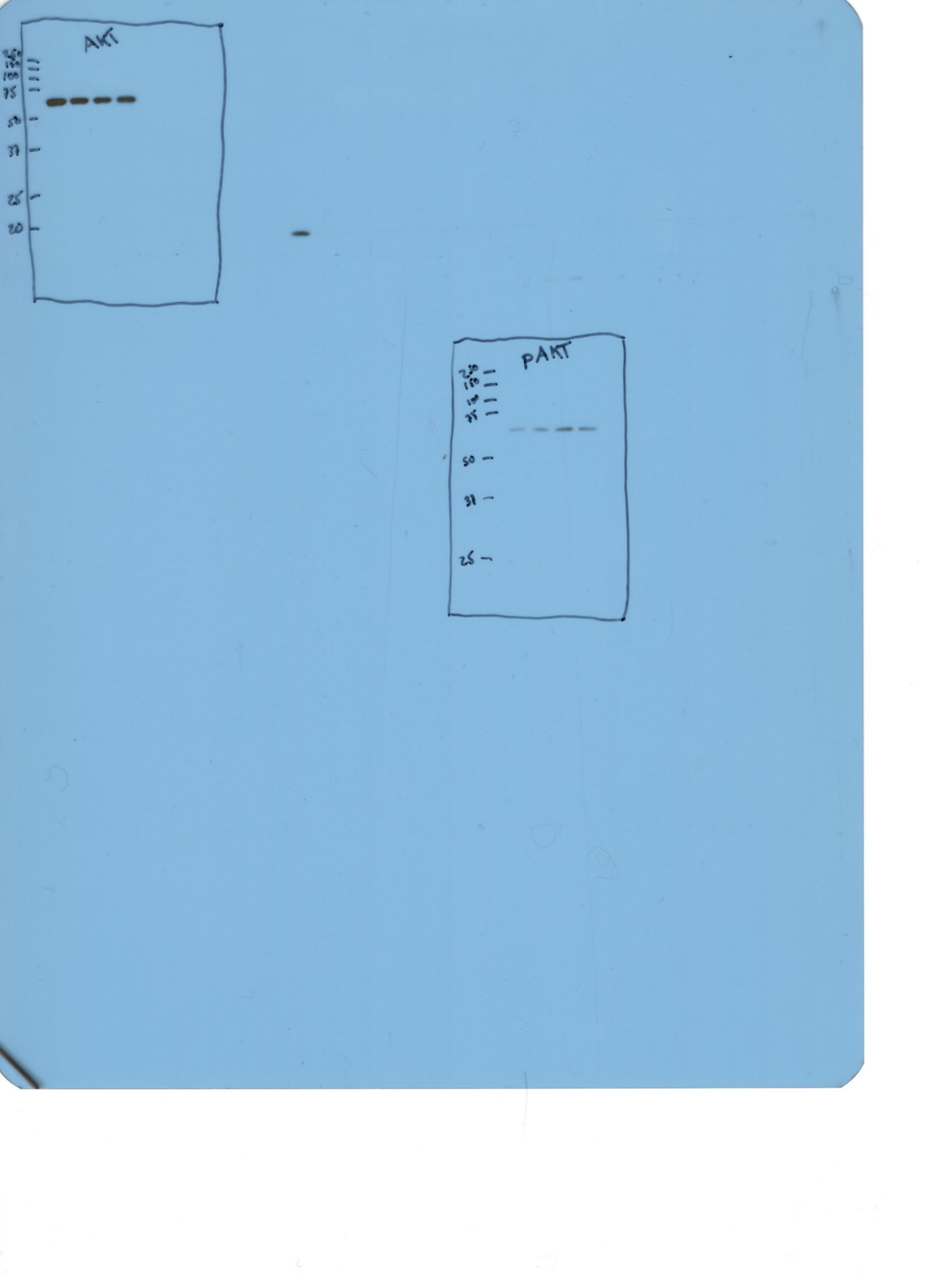


1 2 3 4

AKT

p-AKT

1 2 3 4

Lane 1 : shRNA Ctl 0 min

Lane 2 : shRNA Ctl 30 min

Lane 3 : shRNA Ctl 60 min

Lane 4 : shRNA Ctl 120 min


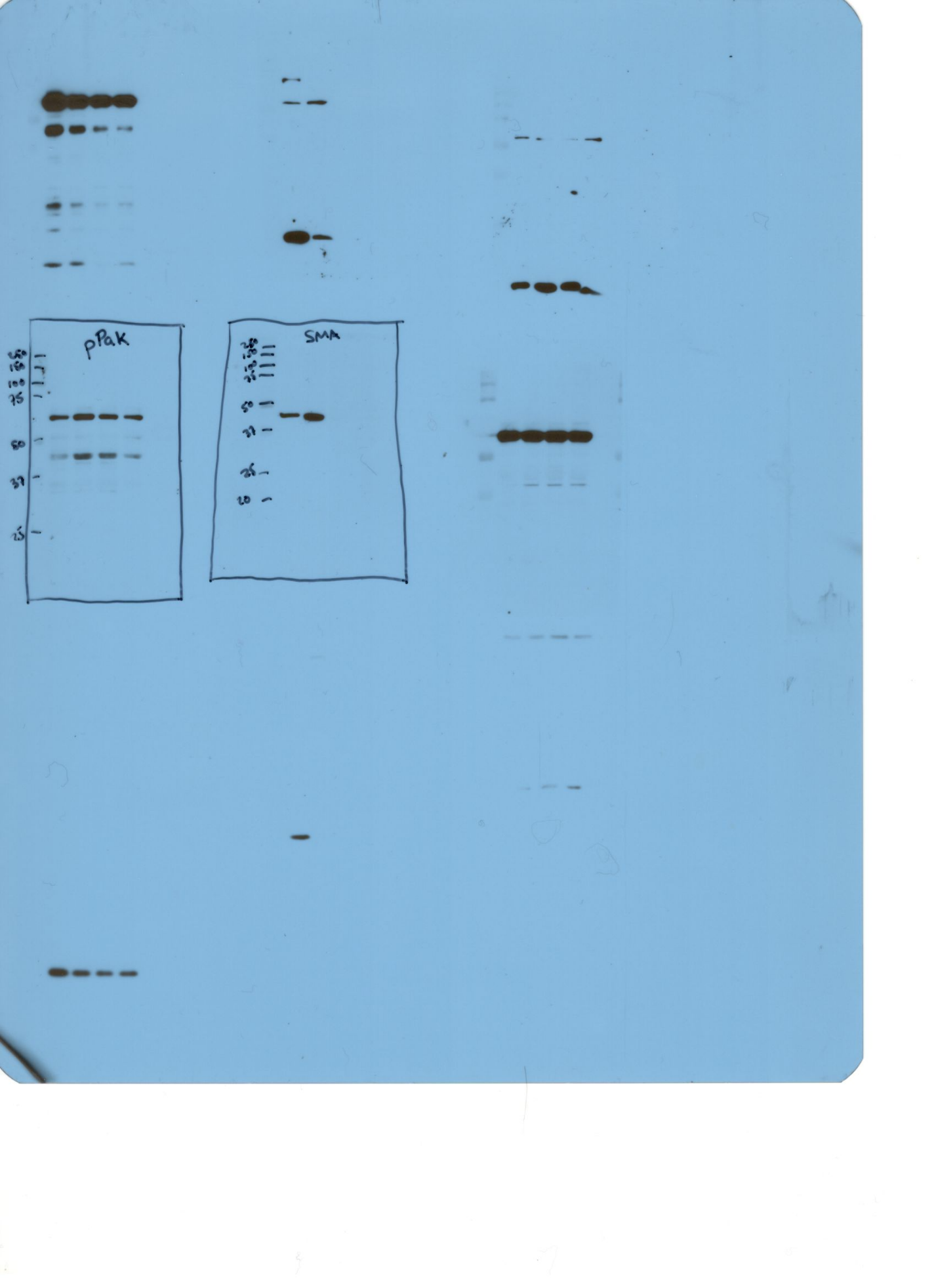


Lane 1 : Rat VSMC

Lane 2 : HAVSMC

Lane 3 : THP-1

Lane 4 : MDA-MB-231

1 2 3 4
